# Comprehensive Structural and Thermodynamic Analysis of Prefibrillar WT α-Synuclein and Its G51D, E46K, and A53T Mutants by a Combination of Small-Angle X-ray Scattering and Variational Bayesian Weighting

**DOI:** 10.1021/acs.jcim.0c00807

**Published:** 2020-08-31

**Authors:** Paolo Moretti, Paolo Mariani, Maria Grazia Ortore, Nicoletta Plotegher, Luigi Bubacco, Mariano Beltramini, Francesco Spinozzi

**Affiliations:** † Department of Life and Environmental Sciences, Polytechnic University of Marche, 60131 Ancona, Marche, Italy; ‡ Department of Biology, University of Padova, 35121 Padova, Veneto, Italy

## Abstract

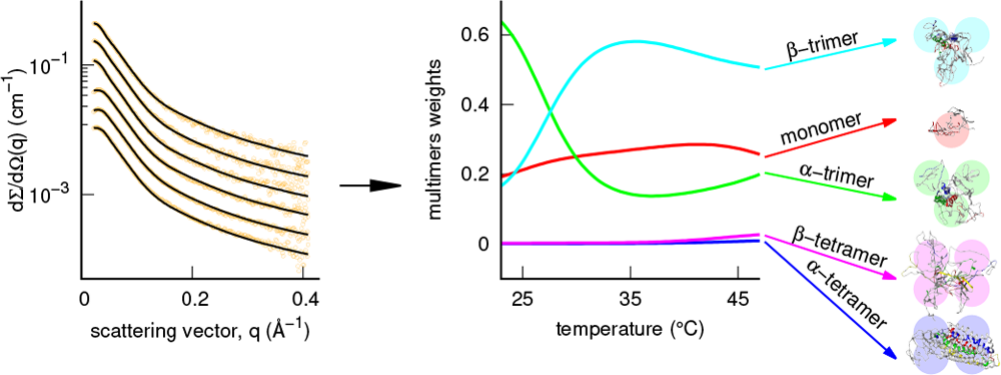

The in solution synchrotron small-angle X-ray scattering SAXS technique has been used to investigate an intrinsically disordered protein (IDP) related to Parkinson’s disease, the α-synuclein (α-syn), in prefibrillar diluted conditions. SAXS experiments have been performed as a function of temperature and concentration on the wild type (WT) and on the three pathogenic mutants G51D, E46K, and A53T. To identify the conformers that populate WT α-syn and the pathogenic mutants in prefibrillar conditions, scattering data have been analyzed by a new variational bayesian weighting method (VBWSAS) based on an ensemble of conformers, which includes unfolded monomers, trimers, and tetramers, both in helical-rich and strand-rich forms. The developed VBWSAS method uses a thermodynamic scheme to account for temperature and concentration effects and considers long-range protein–protein interactions in the framework of the random phase approximation. The global analysis of the whole set of data indicates that WT α-syn is mostly present as unfolded monomers and trimers (helical-rich trimers at low *T* and strand-rich trimers at high *T*), but not tetramers, as previously derived by several studies. On the contrary, different conformer combinations characterize mutants. In the α-syn G51D mutant, the most abundant aggregates at all the temperatures are strand-rich tetramers. Strand-rich tetramers are also the predominant forms in the A53T mutant, but their weight decreases with temperature. Only monomeric conformers, with a preference for the ones with the smallest sizes, are present in the E46K mutant. The derived conformational behavior then suggests a different availability of species prone to aggregate, depending on mutation, temperature, and concentration and accounting for the different neurotoxicity of α-syn variants. Indeed, this approach may be of pivotal importance to describe conformational and aggregational properties of other IDPs.

## Introduction

Intrinsically disordered proteins (IDPs) are a challenge for the biophysical community.^1^ In the past decade, they have in fact attracted attention from both a theoretical and an experimental point of view.^2−4^ In contrast to the structure–function paradigm that has dominated for many years in protein science, it has become clear that the function played by IDPs in many biological processes is due not only to the lack of a unique tertiary structure but also to mainly the high degree of conformational heterogeneity.^5^ The emerging structural picture represents IDPs as an *ensemble* of conformers that transform by a dynamical formation and destruction of secondary structure elements.^6^ This conformational “flexibility” enables IDPs to play a pivotal role in protein–protein recognition, signal transduction, and transcriptional regulation processes.^7^ In addition to these physiological functions, the intrinsic “plasticity” of IDPs has been associated with a number of pathological processes, among which are neurodegenerative diseases and cancer.^8^ IDPs are indeed relatively free to explore a wide conformational landscape and, under certain environmental conditions, they can adopt conformations that trigger aggregation pathways.^9−13^ In the case of cross-β interactions, IDPs progress toward the formation of fibrillar structures, also known as amyloid fibers, which are among the hallmark of several neurodegenerative diseases. Examples are Parkinson’s disease (PD), associated with the fibrillation of α-synuclein (α-syn),^14,15^ Alzheimer’s disease, associated with the β-amyloid (Aβ) peptide, and Hungtington’s disease, in which huntingtin modifications are involved.

One of the main challenging issues in studying IDPs is to describe in a quantitative way the conformational ensemble^3^ in order to identify which are the main structural features that trigger, under diverse chemical–physical conditions, the nucleation step of the fibrillation processes.^3,10^

From a theoretical point of view, molecular dynamics (MD) and molecular mechanics (MM) approaches have been largely exploited to define ensembles of conformers in equilibrium conditions.^16,17^ Recent MD achievements indicate that results strongly depend on the chosen force field and on the model adopted to describe water molecules.^18,19^ Conversely, calculated ensembles of conformers are very often used to interpret sets of experimental data that depend on the distribution of conformational states of the protein. This type of analysis is focused on defining the population weights of each conformer in order to assess its contribution to the averaged data of the observables.^17,20^ Experimental techniques typically analyzed with ensembles of conformers are nuclear magnetic resonance (NMR),^21,22^ small-angle X-ray scattering (SAXS),^7,23^ and Förster resonance energy transfer (FRET) spectroscopy.^1,6^

In all cases, the number of available experimental observables is by far lower than the number of conformers (hence of the degrees of freedom) of the chosen IDP ensembles, and, as a consequence, there is no unique solution which allows one to reproduce the experimental data.^24^ As thoroughly discussed in a review of Ravera et al.,^25^ two opposite approaches have been described to overcome this redundancy problem. The first exploits, in different forms, the maximum entropy principle,^26^ aiming to obtain the least biased probability distribution of each conformer.^24,27^ The second approach is inspired by the “Occam razor’s” rule, i.e., the maximum parsimony principle, which is aimed to determine the minimum number of conformers that are sufficient to recover the experimental data.^20,28−30^

In general, the first approach is considered more suitable to describe the behavior of IDPs, which, being a “natural” ensemble of a great number of conformers, can hardly be imagined as a set of few conformers.^20^ Moreover, the combination of a conformation ensemble with a set of experimental data by means of maximum entropy approaches can be considered a proper way to not only analyze the data but also to validate the theoretical ensembles.

The maximum entropy principle is adopted following two possible strategies. In the first one, it acts directly into molecular simulations by means of restraints between experimental and calculated observables. In the second strategy, the maximum entropy intervenes “a posteriori” as a reweighting method able to determine the weights of the conformers of the ensemble generated by MD or MM simulations in order to optimize the consistency with experimental data.^17,20,31^

The Bayesian formalism,^29,32,33^ which belongs to the maximum entropy scheme, combines prior information on a IDP system with experimental data and, most importantly, takes into account the experimental errors in these data. Hence, the Bayesian inference has been considered particularly suited to investigate IDPs or large intrinsically disordered protein regions (IDPRs).^4,5,34,35^

In particular, a computationally efficient algorithm, called variational Bayesian weighting (VBW), has been adopted to derive the population weights of each conformer together with its standard deviations from NMR data of IDPs, such as α-syn^36^ and Aβ.^37^ The efficiency of VBW is in the use of the simple Dirichlet distribution^38^ to describe both the *prior* probability function of conformers as well as the *posterior* probability that takes into account the information provided by experimental data.

In this article, we first present a novel VBW reweighting method to extensively study the conformational properties of IDPs by taking full advantage of small-angle scattering (SAS) data and their measured variances. The method, which we have called VBWSAS, takes into consideration ensembles of conformers in different multimeric states and applies, for each class of multimers, the VBW strategy within an overall thermodynamic scheme. On the basis of a batch of SAS curves recorded under different chemical–physical conditions, the VBWSAS method is capable of deriving not only the monomer population weights of each multimeric conformer but also their variation as a function of temperature and protein concentration. Also, the secondary structure of IDPs is derived in terms of propensities^39^ of each residue to be in defined regions of the Ramachandran map.

**Figure 1:**
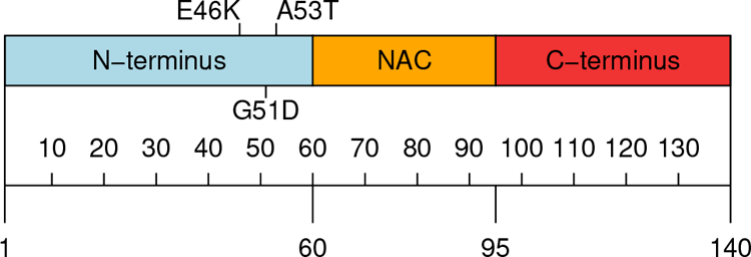
Schematic depiction of α-syn. N-terminus (1–60), NAC domain (61–95), and C-terminus (96–140) are colored light blue, orange, and light red, respectively. Starting and ending residues for N-terminus, NAC region, and C-terminus are labeled.

We then apply the VBWSAS approach to analyze SAXS data of α-syn, a 140 residue protein that constitutes almost 1% of the total proteins in soluble cytosolic brain fractions.^40^ Several different functions have been ascribed to α-syn, including synaptic vesicles trafficking and neurotransmitter release. Coherently, the protein is known to interact with several different binding partners and with negatively charged lipid membranes. A large body of evidence led to the concept that misfolded forms of α-syn are associated with the pathogenesis of Parkinson’s disease (PD).^41^ Under pathological conditions, α-syn forms a heterogeneous ensemble of oligomeric species, some of which are converted to β-sheet-rich fibrillar forms of the protein. These α-syn aggregates have been shown to be toxic for neurons through different molecular mechanisms (reviewed in Plotegher et al.^42^). As depicted in Figure 1, the amino acid sequence of α-syn can be divided into three domains: the N-terminal domain, residues 1–60, which acquire an α-helical structure when the protein interacts with negatively charged lipid membranes or vesicles; the highly amyloidogenic and hydrophobic NAC (non-Aβ-component) domain, residues 61–95; and the C-terminal domain, residues 96–140, enriched in acidic residues and prolines. Both the relatively low hydrophobicity and the high net charge are the cause of the intrinsically disordered nature of α-syn.

Here, we present high-quality synchrotron SAXS data, measured as a function of temperature and concentration, of wild type (WT) α-syn samples as well as three point mutants G51D, E46K, and A53T, associated with the familial form of PD. Temperature and concentration are two parameters already known to impact α-syn aggregation *in vitro*, as well as the pathological point mutations, which were shown to impact on the protein aggregation propensity.^43,44^ The VBWSAS analysis of these SAXS data has been performed by adopting the ensemble of conformers derived by Gurry et al.,^45^ which comprehends unfolded monomers, trimers, and tetramers, these latter in both helical-rich and strand-rich forms. The results and their analyses allow a description of α-syn conformational and multimeric disorder and its changes as a function of pathological point mutation, concentration, and temperature.

## Material and Methods

### The VBWSAS Method

The method here developed considers an *ensemble* of *N* conformers of a IDP under investigation, supposed to be constituted by a polypeptide chain of *N*_aa_ residues (amino acids). We assume that this ensemble contains all the conformational states that monomers of the IDP can adopt in any condition experimentally observed. As a consequence, the IDP molecules will be distributed in *N* conformers according to a set of *monomer population weight*s *w*_*i*_, with the normalization condition ∑_*i*=1_^*N*^*w*_*i*_ = 1. We define **w** as the set of all the monomer population weights. Moreover, we assume that several monomers, in a given conformation, can form defined multimers, so that the ensemble can be subdivided in *M classes of conformers*, which are different for their aggregation number, indicated by *m*. Accordingly, we assume that in the *m*th class of conformers there are *N*_*m*_ conformers, so that ∑_*m*=1_^*M*^*N*_*m*_ = *N*. We introduce the set **W**_*m*_ that contains the *multimer population weight*s within the *m*-class of conformers, with the normalization condition ∑_*j*=1_^*N*_*m*_^*W*_*m*,*j*_ = 1 Hence, if the protein monomer is in the *m*th class of conformers, *W*_*m*,*j*_ represents the multimer population weight in which it is folded according to the *j*th conformer of that class of conformers. The monomer population weight of IDPs in the *m*th class of conformers, i.e., monomers forming multimers with aggregation number *m*, independently on their conformations, is indicated by the symbol ω_*m*_, with the normalization condition ∑_*m*=1_^*M*^ω_*m*_ = 1. With these definitions, the monomer population weight *w*_*i*_ of the *i*th conformer among all the *N* conformers of the ensemble can be written as
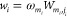
1where *m*_*i*_ is the class of conformers to which the *i*-conformer belongs and *j*_*i*_ indicates which of the  conformers of that class of conformers the *i*-conformer corresponds.

From a thermodynamic point of view, in an ideal solution, the chemical potential of a monomeric chain of the IDP in the *i*-conformer forming a multimer with an *m*_*i*_ aggregation state is defined as
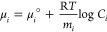
2where R is the universal gas constant, *T* the absolute temperature, and *C*_*i*_ the molar concentration of the *i*-multimer, corresponding to *C*_*i*_ = (*c*/*M*_1_)(*w*_*i*_/*m*_*i*_), *c* being the nominal w/v protein concentration and *M*_1_ the IDP monomer molecular weight. At equilibrium, the chemical potentials of all monomers are equal. Hence, by referring to the first conformer (*i* = 1), at equilibrium we have
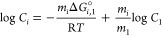
3where Δ*G*°_*i*,1_ = μ°_*i*_ – μ°_1_ is the standard Gibbs free energy change corresponding to the transformation of a solution 1 M of monomers in the 1-conformer and having aggregation number *m*_1_ into monomers in the *i*-conformer with aggregation number *m*_*i*_. The last equation allows one to derive the thermodynamic average, corresponding to the equilibrium conditions, of the monomer population weight in the *i*-conformer, named ⟨*w*_*i*_⟩ , as a function of the one of the first conformer
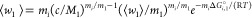
4By combining with the normalization conditions ∑_*i*=1_^*N*^⟨*w*_*i*_⟩ = 1, a polynomial equation of degree γ = max{*m*_*i*_} of the unique variable  is obtainable
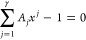
5
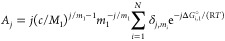
6where δ_*i*,*j*_ is the Kronecker delta function. According to the Abel–Ruffini theorem, analytic solutions are available only up to γ = 4, i.e., up to the formation of tetramers, which are the multimers with the maximum aggregation number in the ensemble adopted in this work.^45−48^ Classical thermodynamics allows one also to describe the standard Gibbs free energy change as a function of *T* in terms of the variations of the standard enthalpy and the standard entropy, both at the reference temperature *T*_0_ = 298.15 K (*ΔS*_*i*,1_^⊖^ and *ΔH*_*i*,1_^⊖^, respectively), and the variation of the heat capacity at constant pressure (*ΔC*_*pi*,1_, supposed not to vary with temperature), all referred to the first conformer, according to . To note, by using eq 7, the ratio , seen in eq 6, can be written in terms of three dimensionless variations of enthalpy, , entropy , and constant pressure heat capacity 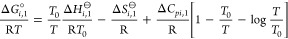
7

### Variational Bayesian Weighting on Different Classes of Conformers

By generalizing the variational Bayesian weighting (VBW) method,^5,8,36,45^ we introduce the *posterior* probability density function (PDF) *f*(**W**_1_, ..., **W**_*M*_) to find out a *M*-dimensional set of multimer population weights, namely, **W**_1_, ..., **W**_*M*_, as
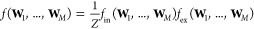
8
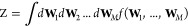
9where *f*_in_(**W**_1_, ..., **W**_*M*_) is the *prior* probability density function, *f*_ex_(**W**_1_, ..., **W**_*M*_) is the *likelihood* probability density function for the experimental observations, and Z is the normalization factor. In this work, experimental observations are SAXS or SANS curves.

For the sake of simplicity and tractability of the problem, we make the strong and crucial assumption that the posterior PDF is factorized in a product of *M* posterior PDFs corresponding to each class of conformers, namely . We also assume that the same assumption holds for the prior PDF . Moreover, according to Fisher et al.,^37^ we make the much stronger assumption that each class of conformers’ PDF can be expressed by a Dirichlet function^38^
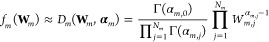
10with Γ(*x*) being the gamma function. To note, the Dirichlet function is fully defined by the set of real positive parameters α_*m*_ ≡ (α_*m,*1_,..., α_*m,N_m_*_), whose sum is defined as . On the basis of the known properties of the Dirichlet distribution, the average and the covariance of the set of multimer population weights are
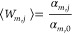
11
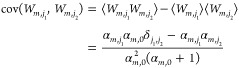
12According to Fisher et al.,^37^ an unbiased prior PDF can be defined through a Dirichlet function with all parameters fixed to α_*m*,*j*_ = 1/2,
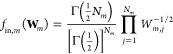
13It is worth noticing that, on the basis of eqs 11 and 12, all the multimer population weights corresponding to the prior PDF have a unique average value ⟨*W*_*m*,*j*_⟩ = *N*_*m*_^–1^, with variance 2*N*_*m*_^–2^(*N*_*m*_–1)/(*N*_*m*_+2).

On these grounds, it can be demonstrated that the sequence of the sets of Dirichlet parameters, **α**_1_, ..., **α**_*M*_, may be found by minimizing the functional
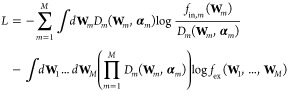
14

### SAS and Variational Bayesian Weighting

The variational Bayesian Weighting formalism can in principle be applied to any experimental observation obtained over a system of IDPs. In the case of a small-angle X-ray or neutron scattering curve (here labeled with a subscript c), the external probability is represented by the following equation
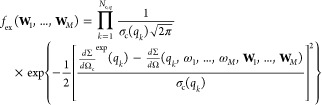
15where  and σ_c_(*q*_*k*_) represent the experimental macroscopic differential X-ray or neutron scattering cross section (SCS) and its standard deviation, respectively, measured in the *k*th of *N*_c,*q*_ values of the scattering vector modulus *q*_*k*_.

In the most general case, let us assume that our investigated IDP, with conformations as well as aggregations described by a selected ensemble, may be at moderate or high concentration, so that in the experimental SAS curve the effect of long-range protein–protein interactions can be observed. Considering a unique average protein–protein structure factor *S*(*q*) that takes into account effective interactions among any conformer or multimers, according to the SAS formalism, the SCS values, which have to be close to the corresponding experimental values provided by SAS, can be expressed as a function of **W**_*m*_ and ω_*m*_
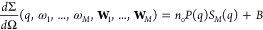
16where the average form factor is
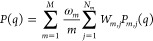
17with  being the total nominal number density of monomers (*N*_*A*_ is Avogadro’s number).

In eq 17,  corresponds to the average, over the polar angles α_*q*_ and β_*q*_ of the scattering vector **q** (orientational average), of the squared form factor of the *j*th conformer of the *m*-class of conformers, a function that can be calculated on the basis of atomic coordinates (e.g., from a PDB file^49^) for both X-rays or neutron scattering by means of methods such as SASMOL.^50^ According to scattering theory, *S*_*M*_(*q*) is the effective or measured structure factor
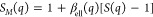
18where the so-called coupling function β_ell_(*q*) is the ratio |⟨*F*_eff_(**q**)⟩|^2^/⟨|*F*_eff_(**q**)|^2^⟩ between the square of the effective orientational average form factor and the orientational average of the effective squared form factor. As discussed by Pedersen et al.,^51^ this function, which typically deviates from 1 for anisometric shapes, can be approximated in an acceptable way by assuming that the effective particle has a simple geometrical shape. In our case, we have considered the shape of a biaxial ellipsoid. The protein–protein structure factor, *S*(*q*), is calculated as the perturbation of the hard sphere structure factor *S*_0_(*q*) obtained with the well-known Percus–Yevick (PY) approximation in the framework of the random phase approximation (RPA).^52^ The perturbation is due to the presence of two Yukawian terms, the first representing the screened Coulumbian repulsion potential and the other an attractive potential.^52^ The relevant parameters of this approximation are “effective values” of the so-called “effective particle”: the number density, *n*, the radius, *R*, the net charge, *Z*, the inverse Debye screening length, κ_*D*_ (which depends on the ionic strength *I*_*S*_ of the protein solution), the attractive potential at contact, *J*, and the range of the attractive interaction, *d*. Considering both the average aggregation number, , and the average of its reciprocal, , these parameters can be approximated as follows: *n* = *n*_◦_⟨*m*^–1^⟩, *Z* = *Z*_1_⟨*m*⟩, *J* = *J*_1_⟨*m*⟩^2/3^, and R = *R*_1_⟨*m*⟩^1/3^, where *Z*_1_, *J*_1_, and *R*_1_ are the monomer net electric charge, the depth of the attractive potential of the monomer, and the average radius of the monomer. Notice that we have supposed that *Z* is simply proportional to ⟨*m*⟩. On the other hand, *J* is supposed to scale as the surface of the protein, here simply defined as the one of the spheres defined by the radius *R*. This latter clearly scales as the cubic root of the volume, which is directly proportional to ⟨*m*⟩. According to this view, the volume of the biaxial ellipsoid, which is used to determine β_ell_(*q*), is (4/3)*πR*_1_^3^⟨*m*⟩; hence, the only parameter that should be optimized is the ellipsoid anisometry ν, i.e., the ratio between the semiaxis *a* and *b*, *b* representing the two equal semiaxes. In eq 16, *B* is a flat background which takes into account incoherent scattering effects, particularly relevant in SANS experiments.

By applying the advantageous properties of the Dirichlet distribution, eq 14 transforms to
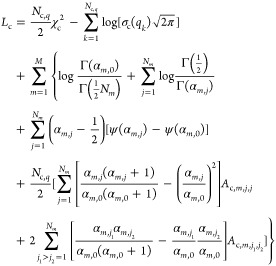
19where χ_c_^2^ is the canonical reduced chi-square, calculated on the basis of the theoretical SCSs (eq 16) corresponding to the thermodynamic averages of all classes of conformers, ⟨ω_*m*_⟩, and multimer population weights, ⟨**W**_*m*_⟩
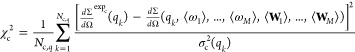
20ψ(*x*) is the digamma function, ψ(*x*) = Γ′(*x*)/Γ(*x*), and the following working pair factors have been introduced
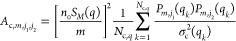
21

It is worth noticing that, on one hand, the factorization of the posterior PDF in a product of *M* posterior PDFs corresponds to the definition of *M* Bayesian problems, each one with its own set of parameters **α**_*m*_. On the other hand, the overall minimization of *L* depends also on the class of conformers weights ⟨ω_*m*_⟩, which are not treated in the Bayesian framework.

Let us now assume that several SAS curves have been measured on the same IDP molecule at different temperatures *T* and total weight concentrations *c*; in this case, a unique analysis of all the data can be realized by combining the thermodynamic model with the VBW approach, with the evident advantage of significantly reducing the number of parameters that should be optimized, hence by increasing the statistical robustness of the achieved results. Indeed, according to the thermodynamic model, through eq 4, we are able to calculate all the values of the monomer population weights ⟨*w*_*i*_⟩, and then, we can derive both the average values of class of conformers parameters,  and, as a consequence, the values of multimer population weights , where *i*_*m*,*j*_ is the conformer among the ensemble of *N* conformers corresponding to the *j*th conformer of the *m*th class of conformers. On the other hand, the Dirichlet parameters can be expressed as a function of ⟨**W**_*m*_⟩ and α_*m*,0_, according to **α**_*m*_ = α_*m*,0_⟨**W**_*m*_⟩. In these conditions, we can minimize an overall functional defined on the basis of all the *N*_c_ SAS curves experimentally available
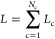
22Adjustable parameters shared by all curves are *ΔH*_*i*,1_^⊖^/(R*T*_0_), *ΔS*_*i*,1_^⊖^/*R*, and *ΔC*_*pi*,1_/*R*, which allow the determination of ⟨*w*_*i*_⟩ at any *T* and *c*, together with the parameters defining the effective structure factor. Curve-specific adjustable parameters are α_*m*,0_.

We have named this new formalism VBWSAS. As shown in the next paragraph, with this approach, we have been able to obtain good quality fits of SAS experimental data.

### Propensities

**Figure 2:**
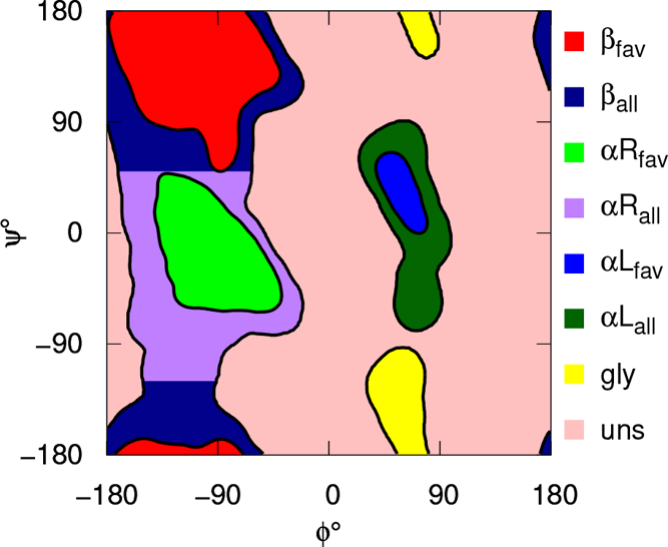
Regions of the Ramachandran map according to the VBWSAS method.

The basic result of the analysis of a set of SAS data of a IDP with the VBWSAS method is the determination, as a function of temperature and protein concentration, of the average monomer population weights ⟨**w**⟩ of the chosen ensemble of conformers. This information allows one to derive other structural features that depend on ⟨**w**⟩. According to Ozenne et al.,^27^ one of the most relevant of this information is the folding *propensity* of each amino acid, defined, in general, as the probability to find the amino acid *a* in an element of the protein secondary structure, such as α-helices or β-sheets. In this framework, it is of relevance to define a criterion to divide the space of the angles ϕ and ψ of the Ramachandran map^53^ in *regions* (*r*) that are well representative of the most significant elements of the secondary structure. For example, according to Ozenne et al.,^27^ the Ramachandran map can be divided into four regions defined as α-left, α-right, β-proline, and β-sheet, a choice which seems unrepresentative to us. Here, we propose to use a different subdivision, based on the distribution ρ(ϕ, ψ) of populated regions in the Ramachandran plot reported by Lovell et al.,^54^ who have analyzed the conformation of 500 high-resolution protein structures through the application of different types of structural analysis. It follows that by contouring the ρ(ϕ, ψ) distribution (normalized to a maximum value of 1) at the levels 0.0005 and 0.02, energetically allowed and energetically favored regions could be identified. Moreover, following Ozenne et al.,^27^ for −180° ≤ ϕ ≤ 0°, the allowed region is subdivided in the α-right allowed region for −120° ≤ ψ ≤ – 50° and in the β allowed region for −180° ≤ ψ ≤ – 120° and 50° < ψ < 180°. As a result, we identify eight regions, which include the three canonical regions of β, α-right, and α-left, each one divided into energetically favorable and energetically allowed, the *glycine zone* and the *unstructured* region. A color-coded visualization of the eight regions in the Ramachandran plot is shown in Figure 2. They are hereafter labeled as β_fav_, β_all_, αR_fav_, αR_all_, αL_fav_, αL_all_, gly, and uns.

We consider now the atomic structure of the *i*-multimer of the ensemble, constituted, for example, by *m*_*i*_ chains. For each *g*-chain and for each *a*-residue (from 2 to *N*_aa_ – 1) of the primary sequence, the ϕ, ψ angles can be calculated, and hence, the index *r*_*i*,*g*,*a*_ of the region of the Ramachandran map to which that residue belongs can be assigned. Clearly, the same residue in the different conformers of the ensemble could match different regions. Hence, considering the average monomer population weights ⟨**w**⟩ screened by SAS experiments, the propensity of the *a*-residue to populate the *r*-region of the Ramachandran map is defined by the following equation
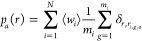
23where, as usual, δ_*i*,*j*_ is the Kronecker’s delta function. The variance of the propensity is
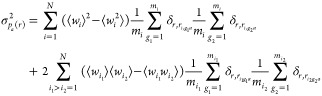
24

### Sample Preparation

Wild type α-syn and the E46K, G51D, and A53T familial mutants were expressed and purified following a previously described protocol.^12^ Briefly, all the α-syn variants were cloned into the pET-28a plasmid and were expressed into the BL21(DE3) *E. coli* strain. Bacterial cultures were grown at 37 °C in Luria–Bertani broth and induced with 0.1 mM isopropyl-*b*-thiogalactopyranoside (IPTG). After 5 h, cells were collected by centrifugation, and recombinant proteins were recovered from the periplasm by osmotic shock. The periplasmic homogenate was boiled for 10 min, and the soluble fraction underwent a two-step (35% and 55%) ammonium sulfate precipitation. The pellet was resuspended in 20 mM Tris-HCl at pH 8.0 and dialyzed. The protein solution was loaded into a 6 mL Resource Q column (Amersham Biosciences) and eluted with a 0–500 mM NaCl gradient. After dialysis against water, all the α-syn variants were lyophilized and stored as powder at −20 °C. For SAXS measurements, proteins were solubilized in water, and ultrafiltration spin columns, with a cutoff of 100 kDa (Amicon), were used to remove larger aggregates, possibly formed during lyophilization and resuspension. Protein purity and integrity were checked after purification and/or storage by SDS-PAGE, and concentration was calculated measuring the absorbance of protein solutions using a spectrophotometer (Perkin Elmer) and considering the molar extinction coefficient of the α-syn at 280 nm equal to 5960 M^–1^cm^–1^.

### SAXS Experiments

Experimental SAXS data were recorded at the BioSAXS beamline BM29 at The European Synchrotron, ESRF in Grenoble (France). The α-synuclein WT and the point mutants G51D, E46K, and A53T were measured at different w/v concentrations *c* comprised between 1 and 10 g/L at temperatures of 25°, 37°, and 45 °C. An automated sample changer was used, and the sample environment was a quartz glass capillary with a diameter of 1.8 mm. The sample-to-detector distance was 2.867 m, and the photon energy was set to 12.5 keV. Accordingly, the modulus of the scattering vector, *q* = 4π sin θ/λ (2θ being the scattering angle and λ = 0.992 Å the X-ray wavelength) was comprised in the range of 0.022–0.41 Å^–1^. Two-dimensional SAXS raw data were recorded by a Pilatus 1 M detector, corrected for detector efficiency, radially averaged to get isotropic signals, and calibrated in absolute units (cm^–1^) by using water. The protein in solution, the buffer, and the empty cell were measured 20 times with an acquisition time of 1 s. The experimental SCS, , of protein samples were obtained by subtracting the signal (averaged over the 20 measurements) from the one of buffer corrected for the protein volume fraction. The experimental standard deviations on SCS, σ_c_(*q*_*k*_), were calculated according to the error propagation theory on the basis of the average values and the standard deviations obtained from the 20 independent measurements of sample, buffer, and empty cell.

## Results and Discussion

**Figure 3:**
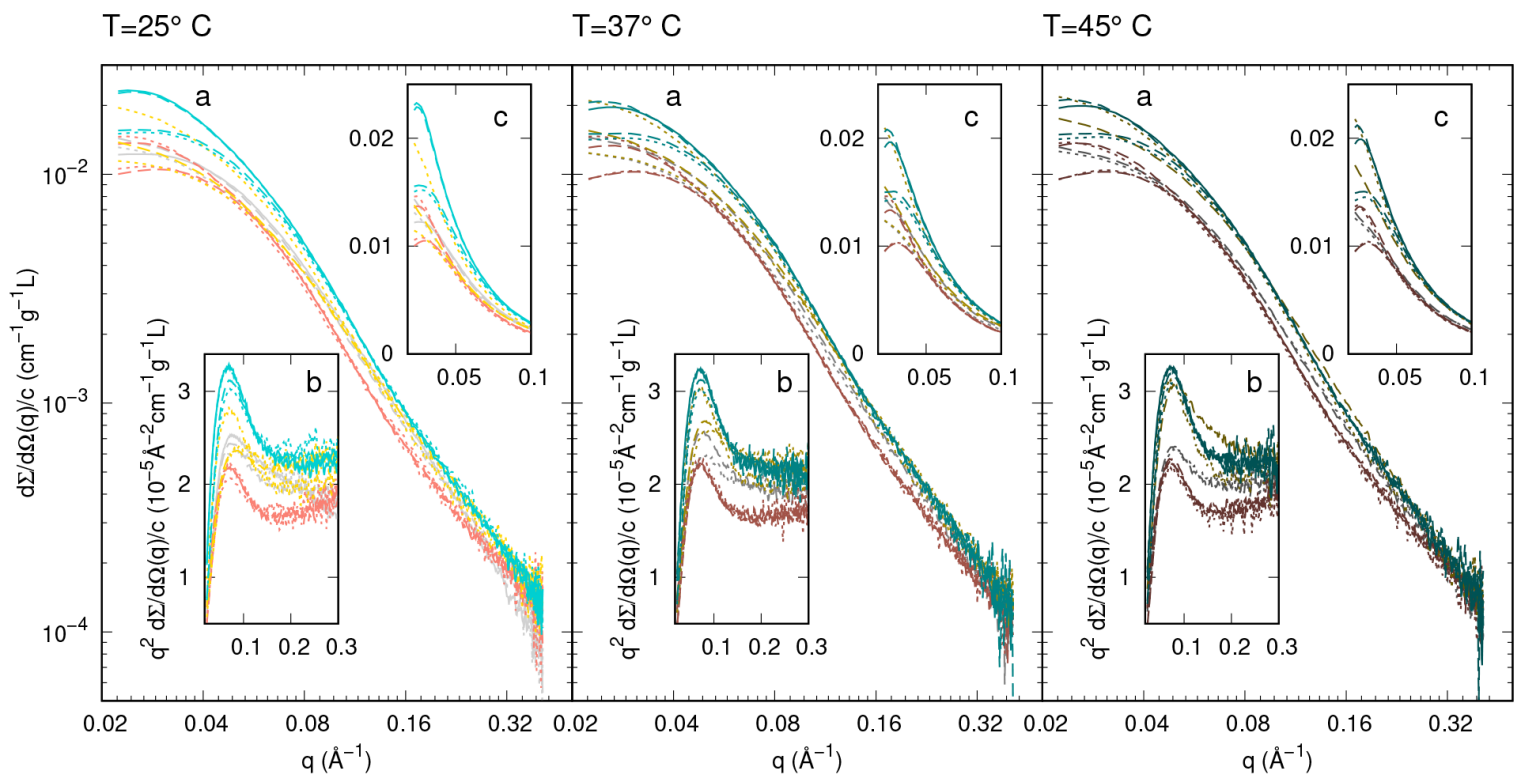
Synchrotron SAXS curves of WT α-syn (gray curves) and the mutants G51D (salmon curves), E46K (gold curves), and A53T (turquoise curves) reported as a function of *q*. Each panel refers to a different temperature, as indicate above. Data, expressed as macroscopic differential scattering cross sections in absolute scale (cm^–1^) divided by the protein w/v concentration *c*, are reported as log–log plots (main panels a), Kratky plots (subpanels b), and linear–linear plots (subpanels c). The darkness of the colors increases with the temperature. Dotted, dashed, and solid lines refer to concentration ranges *c* ≤ 4 g/L, 4 < *c* < 8 g/L, and *c* ≥ 8 g/L, respectively. Error bars have been omitted for clarity.

All measured SAXS curves are reported in Figure 3 in the form of log–log plots (panel a) along the whole *q* range, in the form of Kratky plots (panel b, up to *q* = 0.3 Å^–1^), and as liner-linear plots (panel c) to emphasize the region at low *q*. For the sake of comparison among the various experimental conditions, curves have been divided by the w/v protein concentration *c*. Qualitative similarities among the curves of WT α-syn (gray curves) as well as among the curves of each mutant (G51D, salmon curves; E46K, gold curves; A53T, turquoise curves) can be appreciated. We also observe that, among the curves referring to the same α-syn mutant, the main differences at low *q* (panel c) are due to the presence of a broad interference peak, which changes in position and height mainly, as expected, as a function of *c* (notice that solid curves refer to the highest values of *c*). The Kratky plots (panel b) allow one to better appreciate the differences at high *q* not only among the curves of different α-syn types but also among the ones of the same type. Most importantly, for all protein types, concentrations, and temperatures, Kratky plots show a peak, indicating the presence of folded protein domains, as well as a plateau at high *q*, a signature of unfolded chains,^55^ suggesting that α-syn molecules are either in partially unfolded states or a mixture of folded and unfolded states. Figure 3, panel c, indicates that in the intermediate *q* range around 0.05 Å^–1^, a region of SAXS data that would not be modified by the effect of the structure factor, the normalized curves reach different values, depending on α-syn type, *c*, and *T*. This feature suggests the possible presence of oligomeric forms.

This preliminary and qualitative information has led us to develop the VBWSAS method fully described in The VBWSAS Method section. Indeed, since SAXS data reveal the possible presence of multimeric conformers, it is necessary to adopt an ensemble of protein conformers that includes multimers. On the other hand, the presence of an interference peak at low *q* implies the adoption of a data analysis method that deals not only with form factors but also with structure factors.

On these grounds, we have analyzed with the VBWSAS method the four series of SAXS curves, each series corresponding to one of the four α-syn types. In order to deal with the possible presence of multimers, we have adopted the ensemble of α-syn conformers published by Gurry et al.^45^ This ensemble contains *N* = 189 conformers, recorded as PDB files. To note, these conformers have been selected by the authors, through NMR data, from a larger library of 533 conformers built from a pool of 60,000 structures that, in order to get heterogeneous conformers, was subsequently reduced by a minimum pairwise root-mean-square deviation cutoff of 9 Å. Within the *N* = 189 conformers, there are M = 4 classes of conformers, corresponding to *N*_1_ = 98 monomers (51.9% of the total, referred to as 1A-subclass), *N*_3_ = 15 trimers (7.9%), and *N*_4_ = 76 tetramers (40.2%). Notice that there are no dimers (*N*_2_ = 0). By following the secondary structure assignment proposed by Gurry et al.,^45^ based on the DSSP method,^56^ the trimers are subdivided in *N*_3*B*_ = 4 (2.1%) helical-rich conformers (3B-subclass) and *N*_3*C*_ = 11 (5.8%) strand-rich conformers (3C-subclass). Likewise, among the tetramers, there are *N*_4*D*_ = 19 (10.1%) helical-rich conformers (4D-subclass) and *N*_4*E*_ = 57 (30.2%) strand-rich conformers (4E-subclass). We assume that all these conformers are suitable to define the conformational and multimeric probability distribution of any of the four α-syn types, at any concentration and temperature investigated by SAXS.

**Figure 4:**
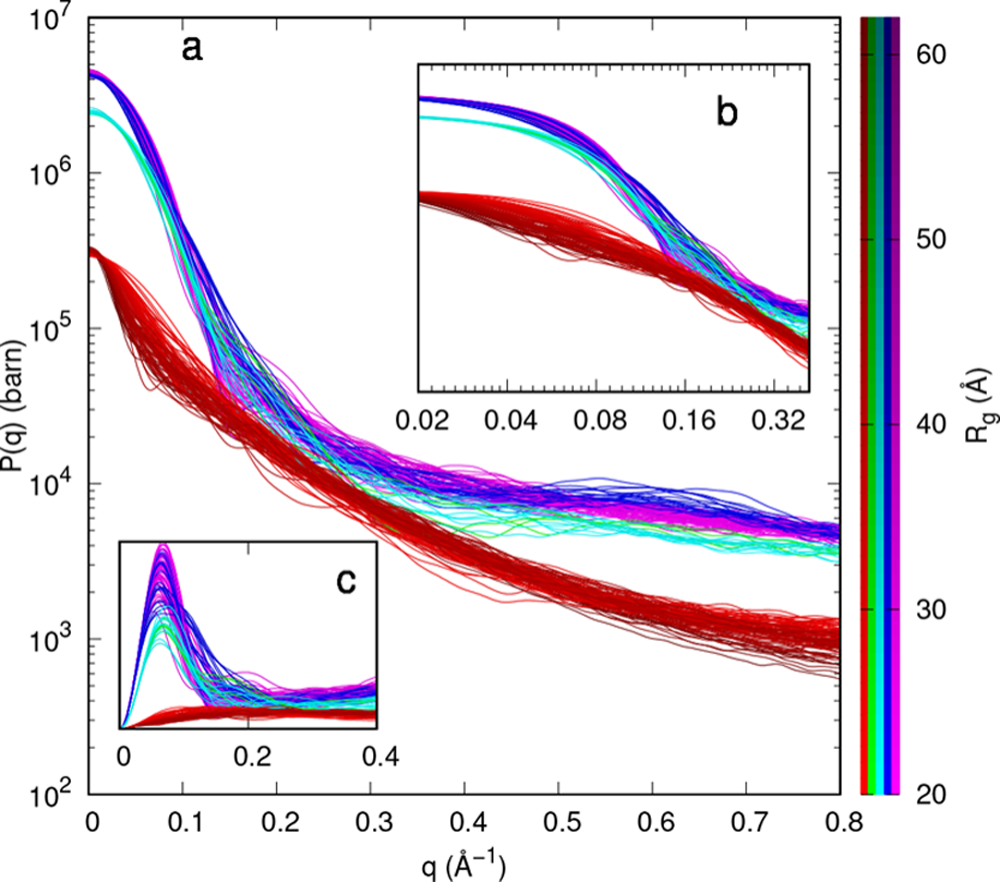
SAXS form factors of the α-syn PDB conformers found by Gurry et al.^45^ calculated with SASMOL. Results are reported as linear-log plots (panel a), log–log plots along the *q*-range of our experiments (panel b), and as Kratky plots (panel c). Red color refers to monomers (1A-subclass). Trimers are shown in green (helical-rich 3B-subclass) and cyan (strand-rich 3C-subclass). Tetramers are shown in blue (helical-rich 4D-subclass) and magenta (strand-rich 4E-subclass). The darkness of the color has been assigned on the basis of the calculated radius of gyration of the conformer, according to the palette on the left. The relative mass density of the hydration water has been fixed to 1.05. See Ortore et al.^50^ for details.

All form factors calculated with SASMOL are shown in Figure 4. Curves in the form of Kratky plots (panel c) clearly show that monomers (red color curves) are unfolded chains, whereas the presence of a peak at *q* ≈ 0.1 Å^–1^ for trimers (green and cyan color curves) as well as tetramers (blue and magenta color curves) confirms that they are folded conformers. We also observed that the behaviors at high *q* (panels a and b) are flatter for folded multimers than for unfolded monomers. These simulations, when compared with the experimental curves shown in Figure 3, suggest that the investigated α-syn types in solution may be seen as mixtures of unfolded monomers and folded multimers, confirming the appropriateness of the Gurry et al.^45^ ensemble adopted by our VBWSAS approach.

VBWSAS has been developed in the Bayesian framework; however, it contains a considerable number of parameters. Hence, in order to obtain robust results, it is worth fixing the value, and whenever possible the *T* or *c* dependencies, of all the parameters that represent either experimental conditions or consolidated chemical–physical properties of our system. Accordingly, considering the thermal expansion of water (see Variation with *T* of Concentration and Solvent SLD, Supporting Information), we have estimated the variation with *T* of the protein w/v concentration and of the scattering length density (SLD) of bulk water.

In the SASMOL method, the contribution of hydration water to the form factor is taken into account by assigning to the water molecules in the first hydration shell a relative mass density *d*_*h*_ different from the one of bulk water. It is known and widely accepted in the SAS community that for folded protein *d*_*h*_ is in the order of 1.05–1.15,^57^ whereas there is not clear evidence of its value for unfolded proteins. We have to consider that, since the volume of the first hydration shell for unfolded proteins is quite large in respect to the dry protein volume, the effect of *d*_*h*_ can greatly vary the form factor. However, unfolded proteins expose toward the solvent both hydrophobic and hydrophilic groups. Hence, it seemed wise to limit the validity range of *d*_*h*_ to 0.95–1.05 and to optimize a unique average value, applied to all the conformers of the ensemble, optimized in the narrow range.

For the screened Coulumbian repulsion potential, we have approximated the value of the relative dielectric constant of the solutions with the one of pure water, whose dependency on temperature is known.^58^ The monomer net charge *Z*_1_ of WT α-syn and of the three mutants G51T, E46K, and A53T has been calculated, according to the primary sequence, as a function of *T*, considering the acidic dissociation constant (p*K*_a_ at the reference temperature *T*_0_;^59^ see Table S1 of the Supporting Information) of the side chain of the 20 amino acids as well as the ones of N- and C-terminal groups. Results are reported in Table S2 of the Supporting Information.

Conversely, since there are not consolidated theories to estimate the parameters of the Yukawian attractive potential (the energy at the contact *J*_1_ and the decay length *d*), we have left them free to vary not only with the α-syn type but also with *T* and *c*. Similarly, we consider free parameters also the average radius of the monomer *R*_1_ (that enters in both the hard sphere and in the two Yukawian terms of the potential) and the ellipsoid anisometry ν defining the coupling function β_ell_(*q*). However, in order to avoid excessive and unlikely oscillations for all these free parameters, a regularization algorithm has been adopted.^60−62^ Therefore, we simultaneously analyze with the VBWSAS method all the SAXS curves measured for each α-syn type by minimizing the following merit function
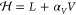
25where *L* is defined according to eq 22, and *V* is the regularization factor
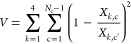
26To note, *V* increases with the difference of the *k*^th^ single curve fitting parameter, *X*_*k*,c_ (*k* = 1, 2, 3, 4 refers to *J*_1_ and *d*, *R*_1_ and ν, respectively), of two close chemical–physical conditions (*c* and *T*), corresponding to the *c*-curve and the *c*′-curve. The constant α_*V*_ in eq 25 is wisely chosen in order to get a factor α_*V*_*V* lower than ≈10% of whole final merit function .

The minimization has been performed by combining the simulated annealing with the simplex methods,^63^ and in order to estimate the standard deviations of all fitting parameters, it has been repeated several times by randomly sampling each point of the SAXS experimental curves from a Gaussian with mean value  and variance σ_c_^2^(*q*_*k*_).

**Figure 5:**
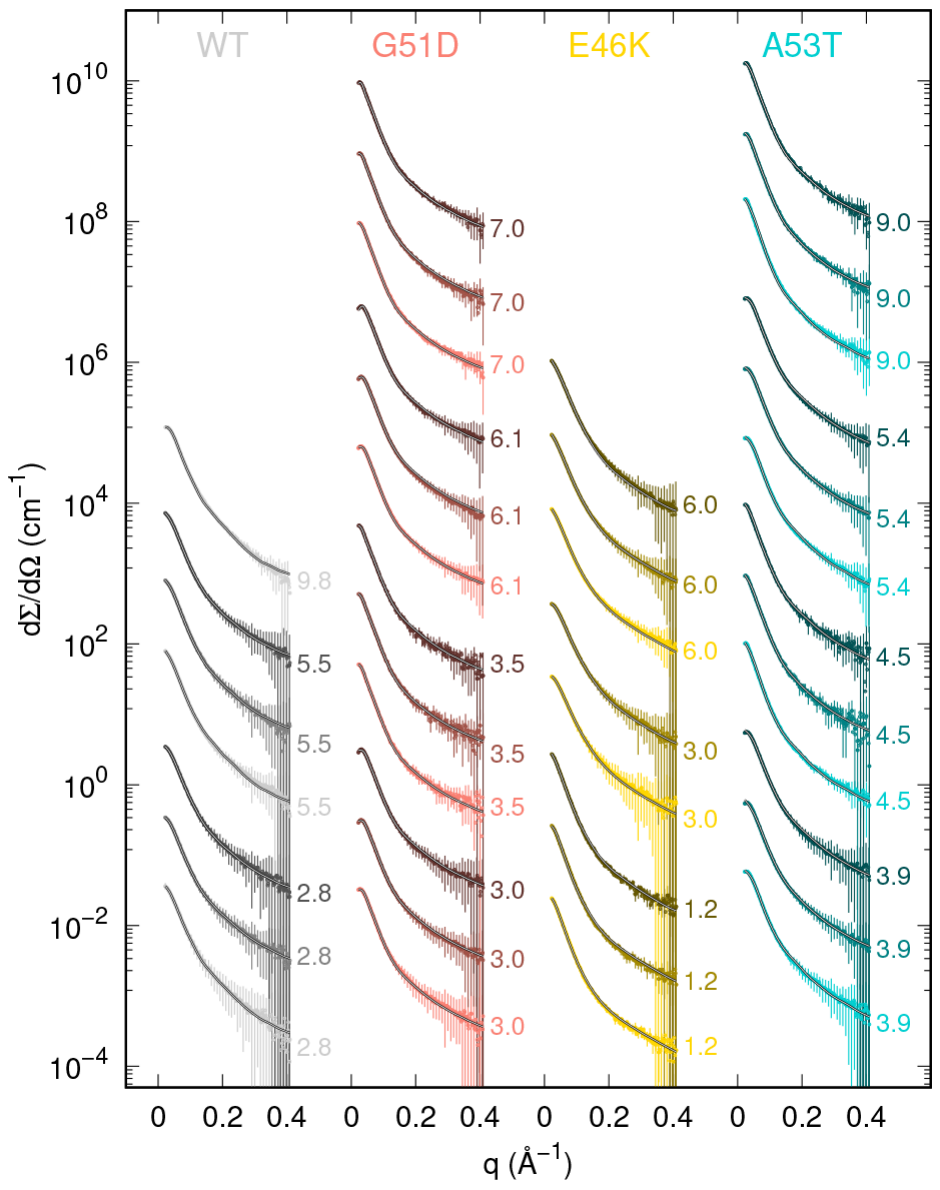
Experimental SAXS curves of WT α-syn and the mutants G51T, E46K, and A53T superimposed with the best fits obtained with VBWSAS (solid black and white lines). Curves are color coded on the basis of the α-syn type and of the temperature, according to the caption of Figure 3. The nominal protein concentration is reported beside each curve in g/L units. For the sake of a better visualization, curves in the same column, referring to the α-syn type shown on the top of the column, have been staked by multiplying for a factor 10^*m*–1^, *m* being the index of the row from the bottom. Experimental standard deviations are reported as error bars at every 5 points, for clarity.

Best fitting curves obtained by applying VBWSAS for each of the four series of SAXS curves are reported in Figure 5. To note, we have also performed VBWSAS analyses by using subsets of the ensemble of Gurry et al.^45^ For all α-syn species, we have found that the best curve fits, in particular at low *q*, are obtained by using all the 189 conformes of the Gurry et al.^45^ ensemble, confirming the appropriateness of its structural heterogeniety. Detailed graphs reporting the distinct contributions of form and structure factors are shown in Figures S1–S4 of the Supporting Information. We notice that all the experimental features of SAXS curves at both high and low *q*, including the interference peak mainly evident at the largest concentration, are nicely reproduced by VBWSAS. It is also worth considering that we have fully exploited the absolute calibration of the data and the very precise buffer subtraction procedure described in the SAXS Experiments section.

**Figure 6:**
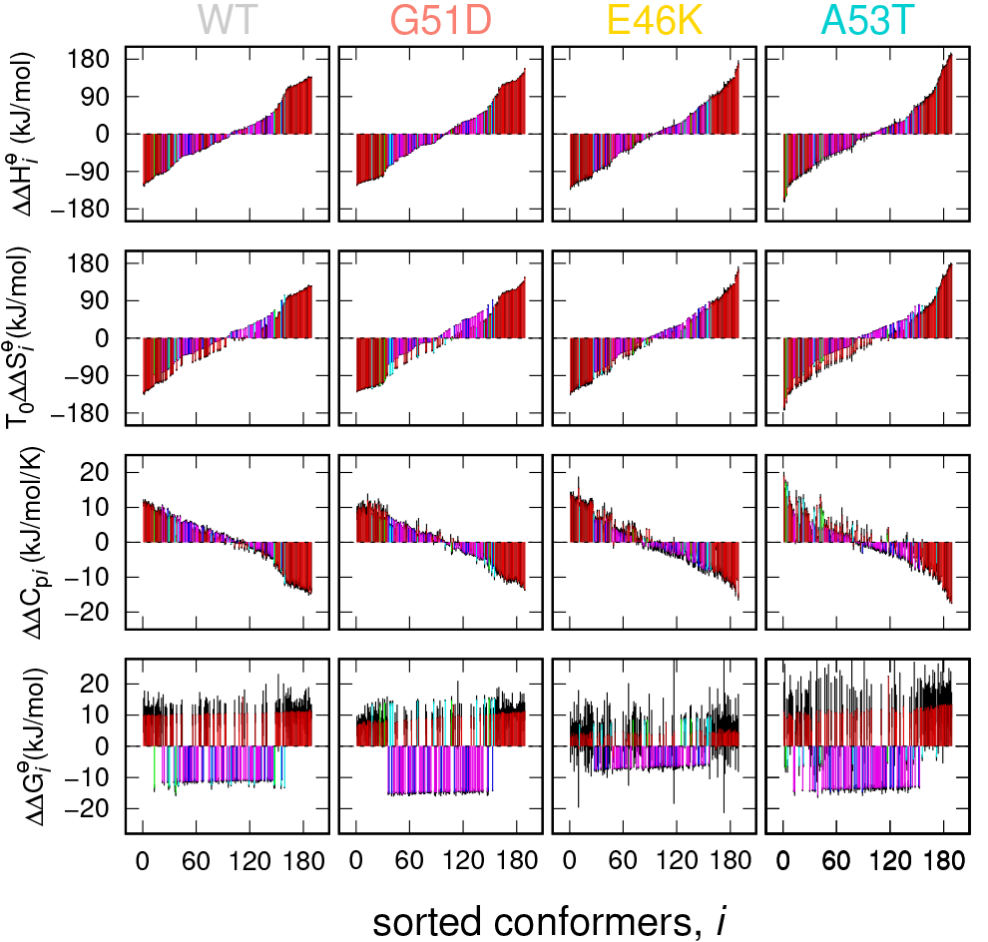
Thermodynamic fitting parameters obtained by the analysis of SAXS data with VBWSAS of WT α-syn and the mutants G51T, E46K, and A53T. All data are reported as differences with respect to their mean (ΔΔΦ_*i*_ = ΔΦ_*i*,1_ – ⟨ΔΦ⟩). The 189 conformers are sorted on the basis of the standard enthalpy changes reported, for each α-syn type, in the top panels. The same sorted series of conformers is adopted in the other panels regarding the same α-syn type, which report the variation of standard entropy, heat capacity, and standard Gibbs free energy (this latter calculated by *ΔΔH*_*i*_^⊖^ – *T*_0_*ΔΔS*_*i*_^⊖^). The color hue of the histogram bars is assigned according to the α-syn subclass (1A red, 3B green, 3C cyan, 4D blue, 4E magenta), and the darkness of the color increases with *R*_*g*_, as described in the caption of Figure 4. Standard deviations are shown as black error bars.

We look now at the results, starting from the thermodynamic fitting parameters of each *i*-conformer reported, in the form of histograms, in the panels of Figure 6 and calculated as differences with respect to the mean value, ΔΔΦ_*i*_ = ΔΦ_*i*,1_ – ⟨ΔΦ⟩, ΔΦ representing *ΔH*_*i*,1_^⊖^, *T*_0_*ΔS*_*i*,1_^⊖^, *ΔC*_*pi*,1_, and *ΔG*_*i*,1_^⊖^ = *ΔH*_*i*,1_^⊖^ – *T*_0_*ΔS*_*i*,1_^⊖^. It should be noticed that the histogram bars have been colored on the basis of the subclass and the radius of gyration of the *i*-conformer, following the same color settings of Figure 4. To simplify the interpretation of these results, we have sorted the *N* = 189 conformers in ascending order of *ΔΔH*_*i*_^⊖^, as reported in the top panels of Figure 6. Moreover, in the other three panels below each enthalpy panel, related to the same α-syn type, we report the data as a function of the same sorted sequence of conformers used in the enthalpy panel. In this way, we can better estimate the relationships, if any, between the thermodynamic parameters of each *i*-conformer. The high similarity between the first two panels of the same column of Figure 6 clearly shows an entropy/enthalpy compensation effect.^64^ Indeed the variations *ΔΔH*_*i*_^⊖^ and *T*_0_*ΔΔS*_*i*_^⊖^ are comprised between −100 and 100 kJ/mol, whereas their difference, corresponding to *ΔΔG*_*i*_^⊖^ (bottom panels of Figure 6), is lower by nearly 1 order of magnitude, varying from −10 to 10 kJ/mol. These low free energy differences, close to the thermal energy at room temperature, confirm that the chosen ensemble of conformers is suitable to describe a disordered conformational landscape. Interestingly, *ΔΔG*_*i*_^⊖^ of monomers and, in less extent, trimers (1A, 3B, and 3C subclasses shown with red, green, and cyan bars, respectively) are mostly positive, whereas for the tetramers subclasses (4D and 4E, blue and magenta bars, respectively) they are negative.

**Figure 7:**
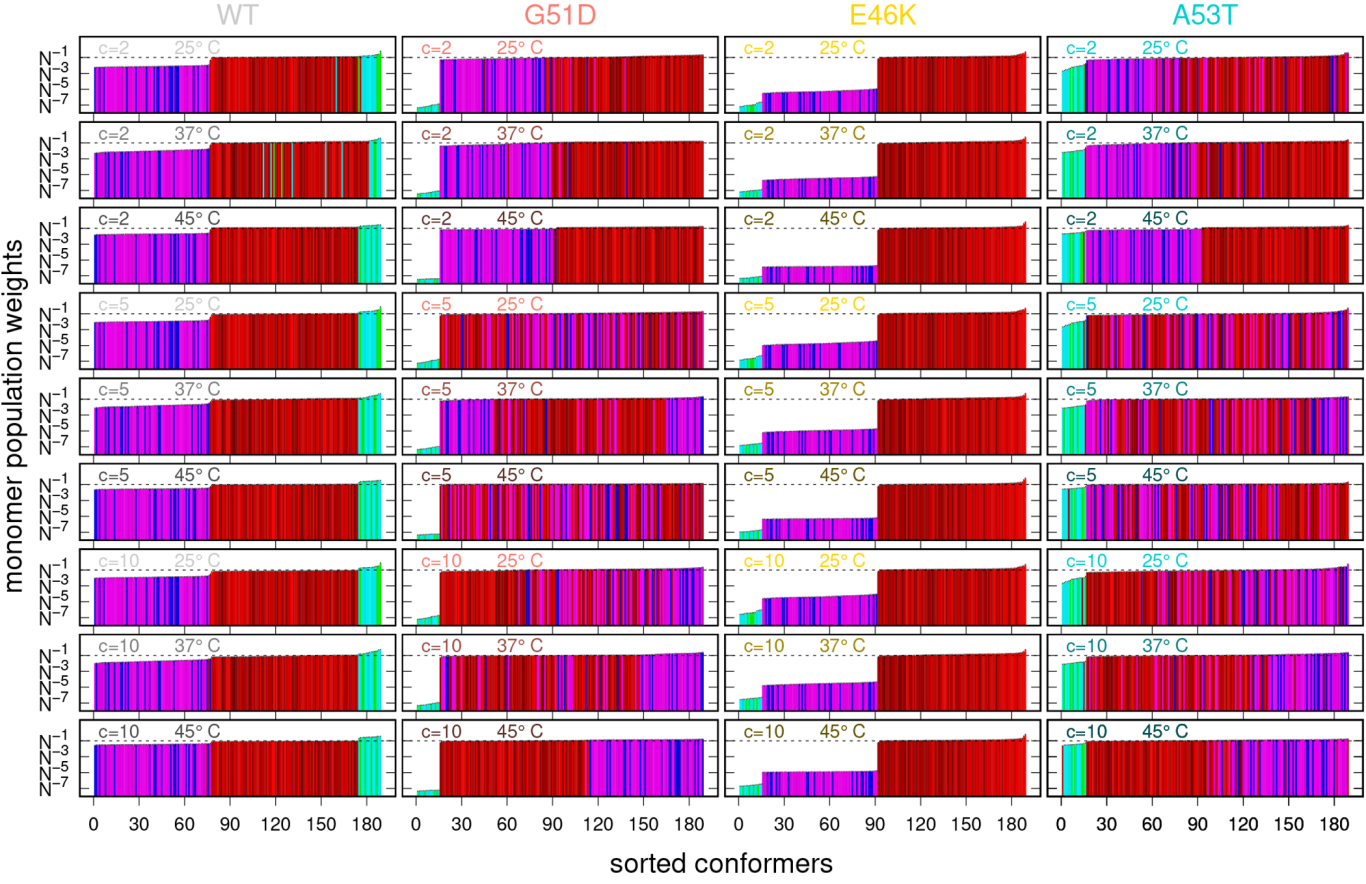
Histograms of the average monomer population weights ⟨*w*_*i*_⟩ associated with the *N* = 189 α-syn PDB conformers exploited by VBWSAS for the best fit analysis of the four batches of SAXS curves for WT α-syn and the three mutants G51T, E46K, and A53T shown in Figure 3. Values are calculated from eq 4 on the basis of the fitted values of all the thermodynamic parameters (Figure 6). Results are sorted from the lowest (left) to the highest (right) ⟨*w*_*i*_⟩. The color hue of the histogram bars is assigned according to the α-syn subclass (1A red, 3B green, 3C cyan, 4D blue, 4E magenta) and the darkness of the color increases with *R*_*g*_, as described in the caption of Figure 4. Standard deviations are shown as black error bars.

In the VBWSAS method, the fitted thermodynamic parameters are used to calculate the average monomer population weights ⟨*w*_*i*_⟩ (eq 4), which clearly represent the most relevant information regarding the conformational landscape. In order to provide a comprehensive description of the achieved results, we have calculated them, for each α-syn type, at three unique values of w/v concentration (2, 5, and 10 g/L) and three unique values of temperature (25°, 37° and 45 °C). Results are reported in Figure 7 in the form of histograms, with bars colored according to the same code used in Figure 6 and with the *i*-conformers sorted in ascending order of ⟨*w*_*i*_⟩.

We consider first the panels relative to WT α-syn. The proximity of the bars with the same hue of color is immediately evident, as well as the similarity of their heights. This result deserves a more thorough consideration, bearing in mind that it has been obtained by analyzing our experimental SAXS data, at different *c* and *T*, and adopting an ensemble of conformers already filtered through the NMR experiments reported by Gurry et al.^45^ We observe that WT α-syn molecules are mostly present as trimers (3B and 3C subclasses, green and cyan bars, respectively), closely followed by all the conformers in the monomeric state (1A, red bars) and with the remaining tetramers (4C and 4D, blue and magenta bars, respectively) in the last positions. Moreover, we see that the bars of trimers and monomers reach a value quite close to *N*^–1^ (dashed line in Figure 4, corresponding to the totally flat (unbiased) monomer population weight distribution), and the tetramers’ bars arrive at values slightly higher than *N*^–2^. Basically, despite these differences, the VBWSAS analysis of WT α-syn confirm that all the *N* = 189 conformers of Gurry et al.^45^ significantly contribute to describing the conformational distribution.

**Figure 8:**
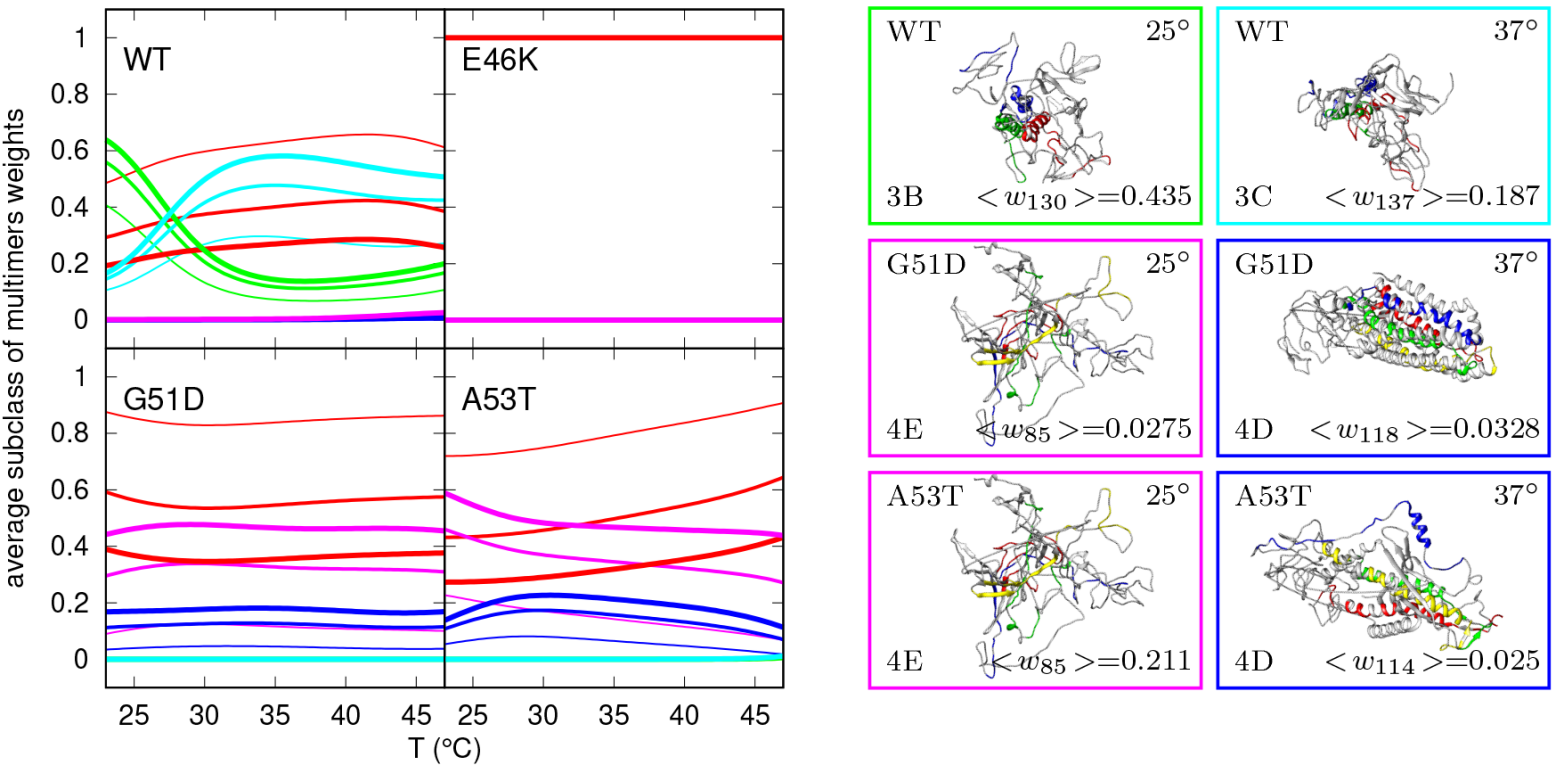
Left panels show temperature trends of the subclasses of conformers ω_*m*_ with *m* = 1A (monomer, red), *m* = 3B (helical-rich trimers, green), *m* = 3C (strand-rich trimers, cyan), *m* = 4D (helical-rich tetramers, blue), and *m* = 4E (strand-rich tetramers, magenta) calculated from the thermodynamic parameters found by the VBWSAS analysis of SAXS data for WT α-syn and the three mutants G51T, E46K, and A53T. Thin, regular, and thick lines refers to *c* = 2 g/L, *c* = 5 g/L, and *c* = 10 g/L, respectively. Right panels show the most populated conformers at *c* = 10 g/L, of all α-syn types apart from E46K. On the bottom left and bottom right of each conformer, the subclass and the obtained ⟨*w*_*i*_⟩ (written up to the last significant digit) are reported, respectively. The eight sequences that, according to Guerrero-Ferreira et al.,^65^ are forming the parallel β-strands in the fibrils (i.e., residues 42–46 (β1), 48–49 (β2), 52–57 (β3), 59–66 (β4), 69–72 (β5), 77–82 (β6), 89–92 (β7), and 94–102 (β8)) and that belong to the different monomers in the trimers and in the tetramers are shown with different colors.

We also observed that, with increasing *T*, the heights of the bars get closer in value. The *T* effect, as well as the less pronounced concentration effect, are better visualized in Figure 8 (left panel), which reports the *T*-trends of the subclasses of conformers ω_*m*_ (which is the sum of ⟨*w*_*i*_⟩ for *i* belonging to the same subclass) at the three selected values of *c*. We see that, for WT α-syn (Figure 8, top left panel), the trends of the monomers subclass weights (ω_1A_, red curves) show a maximum at ≈42 °C, which depends on *c* going from ≈0.7 at 2 g/L to ≈0.3 at 10 g/L. We also see that by increasing *T* the ω_3B_ weights (helical-rich trimers, green curves) decrease, whereas ω_3C_ (strand-rich trimers, cyan curves) increases, and this effect has a direct correlation with protein concentration. Interestingly, at high concentration (5–10 g/L), a α/β transition is observed: up to ≈27 °C the most populated subclass is 3B (helical-rich trimers) and subsequently the 3C (strand-rich trimers) subclass, which reaches a maximum concentration at ≈35 °C. In Figure 8 (top right panels), the most populated conformers at 10 g/L and for *T* = 25 °C and *T* = 37 °C are represented; we notice the proximity of the sequences of residues, belonging to the three different chains, associated with the formation of fibrils, as reported by Guerrero-Ferreira et al.,^65^ suggesting that these trimeric conformers might be representative of those that trigger the nucleation in the fibrillation processes. A more detailed visualization of the most populated conformers, at 10 g/L and for *T* = 25 °C and *T* = 37 °C, is shown in Figure S5 of the Supporting Information. The obtained results, regarding the temperature effects, are in agreement with the increase of nucleation and growth of fibrils, as reported by Morris and Finke.^66^ In terms of protein concentration effects, the results reported here confirm the aggregation propensity reported by many *in vitro* studies (reviewed in Plotegher et al.^42^). Finally, the effects reported are relevant for the pathogenesis of synucleinopathies, considering for instance that duplication and triplication of the gene encoding for α-syn and the associated increase α-syn concentration cause inherited forms of early PD onset.^67^

We turn now to describe the results obtained for the G51D α-syn mutant type. The average monomer population weights ⟨*w*_*i*_⟩ are shown in Figure 7, second column of panels. The changes with respect to WT α-syn are evident: the trimers (green and cyan bars) go in the last positions, with values of ⟨*w*_*i*_⟩ in the order of *N*^–7^, whereas tetramers (blue and magenta bars) and monomers (red bars) show values of ⟨*w*_*i*_⟩ of the same order of magnitude. Moreover, by observing the top and the bottom panels (corresponding to the more distant conditions in terms of *c* and *T*), we see that at low concentration and temperature the monomers have the highest weights, whereas tetramers overcome monomers at the highest values of *c* and *T*. We also observed a more mixed situations in the intermediate panels, corresponding to the other combinations of *c* and *T*. The same behavior can also be detected in the correlation map of ⟨*w*_*i*_⟩, calculated for the same *c* and *T* conditions, between WT and the G51D α-syn mutant type and reported in Figure S6 of the Supporting Information. This result is also confirmed in Figure 8, bottom left panel. The values of ω_1A_ (red curves) are almost independent of *T* and change from ≈0.8 at 2 g/L to ≈0.4 at 10 g/L, and in parallel, the subclasses of conformers of the strand-rich tetramers, ω_4E_ (cyan curves), arrives to ≈0.6 at 10 g/L, being ≈0.1 at 2 g/L. Figure 8 (middle right panels) show the most populated conformers, at 10 g/L, for *T* = 25 °C and *T* = 37 °C, evidencing a possible role of compact helical-rich tetramers in the fibrils nucleation at high temperature.

A completely different landscape has been defined by VBWSAS for the E46K mutant. The E46K panels of Figure 7 provide a simple message; only the monomeric conformers (red bars) are significantly present in solution, independently on *c* or *T*. We see, in fact, that the tetramers ⟨*w*_*i*_⟩ values (blue and magenta bars) are very low, around *N*^–5^, and those of the trimers even lower, ≈ *N*^–7^. This result is fully confirmed in the top right panel of Figure 8, where we just observed ω_1A_ = 1. Looking carefully at the shades of the red bars in the E46K panels of Figure 7, it can be notice that, particularly at low *c* and high *T*, the brightest bars are in the first positions, suggesting the prevalence of monomers with the lowest gyration radii. A similar observation is confirmed in the E46K correlation maps of ⟨*w*_*i*_⟩ shown in Figure S6 of the Supporting Information.

Finally, we analyze results for the A53T α-syn mutant type. Corresponding panels of Figure 7 show the most marked variability of the results in respect to *c* and *T*. We see, in fact, that at *c* = 2 g/L monomers (red bars) prevail, followed by tetramers (blue and magenta bars) and, to a lesser extent, by trimers (green and cyan bars), and this trend is reinforced with increasing *T*. The situation is less straightforward at *c* = 5 g/L, where the populations of monomers and tetramers are close. At *c* = 10 g/L, in particular at the highest temperatures, the prevalence of tetramers with respect to monomers is evident. It can be noticed that the values of ⟨*w*_*i*_⟩ for trimers markedly increase with *T*, for any value of *c*. Once more, the bottom left A53T panel of Figure 8 confirms this monomer–tetramer competition. Regarding the most populated conformers at 10 g/L for *T* = 25 °C and *T* = 37 °C (Figure 8 right A53T panels), it emerges that at high *T* the predominant tetrameric helical-rich conformers are less compact than those of the G51D mutant under the same *c* and *T* conditions, with the sequences of residues responsible of the fibril formation (show in blue, red, green, and yellow colors) quite far apart. The A53T correlation maps of Figure S6 of the Supporting Information confirm this monomer–tetramer competition and add the information that strand-rich tetramers (magenta curves or symbols) dominate with respect to helical-rich tetramers (blue curves or symbols).

These results are in agreement with the information available in the vast literature on the aggregation kinetics of the different mutants compared to WT.^44^ Specifically, the G51D and A53T mutants, which, according to our results, show an increased proportion of β-strand multimeric species, have been reported to be more prone to aggregation than WT α-syn. In addition, it has been revealed that the E46K mutant shows a longer lag phase, suggesting that the nucleation centers that trigger the aggregation are scanty when compared to the other mutants; this observation is fully confirmed by the VBWSAS results on E46K, which indicate that only monomeric conformers have a significant population. Aggregation kinetics often present some variability and reproducibility issues that also depend on the method used to measure the process. Results obtained by using VBWSAS to weigh the ensemble of monomers and multimers for the three G51D, A53T, and E46K mutants under different concentration and temperature conditions may help in rationalizing experimental results previously found and in carefully planning new experiments.

All previous evaluations about the properties of ⟨*w*_*i*_⟩ for the investigated α-syn type reflect the behavior of form factors (eq 17). However, SAXS curves also contain information about the structure factors, which are enclosed in the VBWSAS formalism (eq 18). Fitting parameters related to the structure factors obtained by the analysis of all the experimental curves shown in Figure 5 are detailed reported, as a function of *T* and for selected ranges of *c*, in Figure S8 of the Supporting Information. The high error bars obtained for most of these parameters suggest that the information content regarding protein–protein interaction, extracted from the whole *q* range of our experimental curves, should be considered quite low. Hence the physical interpretation of these parameters should be taken with a word of caution. For example, the mean radius of the monomer *R*_1_, calculated over all the *c* and *T* values of each α-syn type, results 24 ± 1 Å for WT, a value that decreases to 14 ± 1 Å for G51D and becomes much lower for E46K (8.1 ± 0.6 Å) and A53T (9 ± 1 Å). We recall that those values are subsequently multiplied by the average aggregation number ⟨*m*⟩ to get the radius of the hard-sphere term in the pair potential and the value ⟨*m*⟩, being a function of ⟨*w*_*i*_⟩, change with *c* and *T*, as shown in Figure S9 of the Supporting Information. Considering the attractive potential at contact, written as *J* = *J*_1_ ⟨*m*⟩^2/3^, fitted values of *J*_1_ for WT α-syn and for the two mutants G51D and A53T are almost constant with *c* and *T*. Their mean values are 390 ± 40, 91 ± 7, and 110 ± 20 kJ/mol, respectively. The case of E46K is different; *J*_1_ changes from 500 ± 10 kJ/mol at low concentrations to 240 ± 80 kJ/mol at intermediate concentrations, suggesting that the E46K monomers experience a more complex network of interactions quite sensitive to *c* variations.

**Figure 9:**
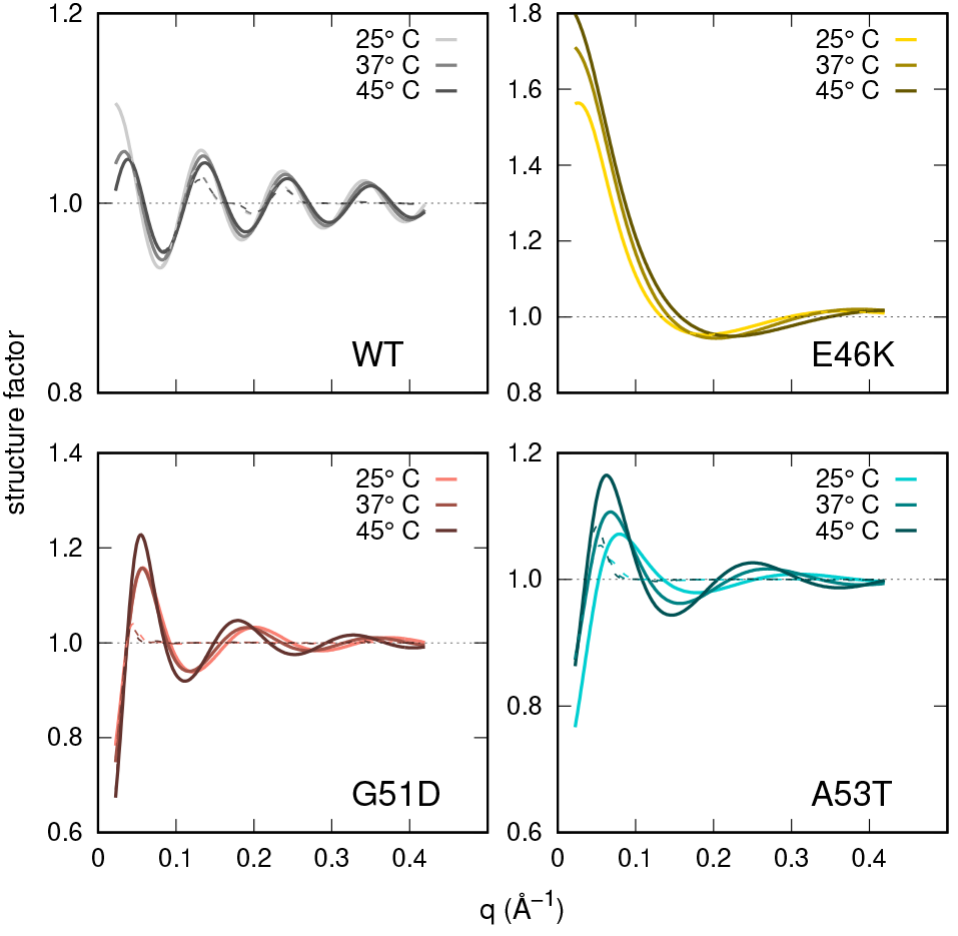
Structure factors, *S*(*q*) (solid lines), and measured structure factors, *S*_*M*_(*q*) (dashed line), (eq 18) obtained by SAXS data analysis with VBWSAS for WT α-syn (*c* = 5.5 g/L) and three mutants G51T (*c* = 6.1 g/L), E46K (*c* = 6.0 g/L), and A53T (*c* = 5.4 g/L) at the three temperatures as shown in the legends. Detailed parameters are listed in Table S3 of the Supporting Information.

An overall evaluation about the structure factors is provided in Figure 9, where both functions *S*(*q*) (solid lines) and *S*_*M*_(*q*) (dashed lines) are reported for the four α-syn types at the intermediate *c* and for different *T*. We notice that for WT, G51D, and A53T types these functions slightly oscillate around 1, indicating a competition between attractive and repulsive forces, whereas the E46K type shows a different regime, with structure factors markedly higher than 1 at low *q*, indicating a prevalence of attractive forces. Moreover, it should be underlined that for both G51D and A53T α-syn types, *S*_*M*_(*q*) is quite damped with respect to *S*(*q*), an effect due to the features of the coupling function β_ell_(*q*) (eq 18) that mainly depend on ⟨*m*⟩, always greater than 2 for G51D and A53T α-syn types, and the ellipsoid anisometry ν, which is ≈5 for both of them (Figure S8 of the Supporting Information). On the contrary, ν is ≈2 for WT and E46K α-syn types, and for the latter, the conformers are substantially monomers (Figure S9 of the Supporting Information). Hence, the effect of β_ell_(*q*) is less marked, and no differences between *S*(*q*) and *S*_*M*_(*q*) are observed.

**Figure 10:**
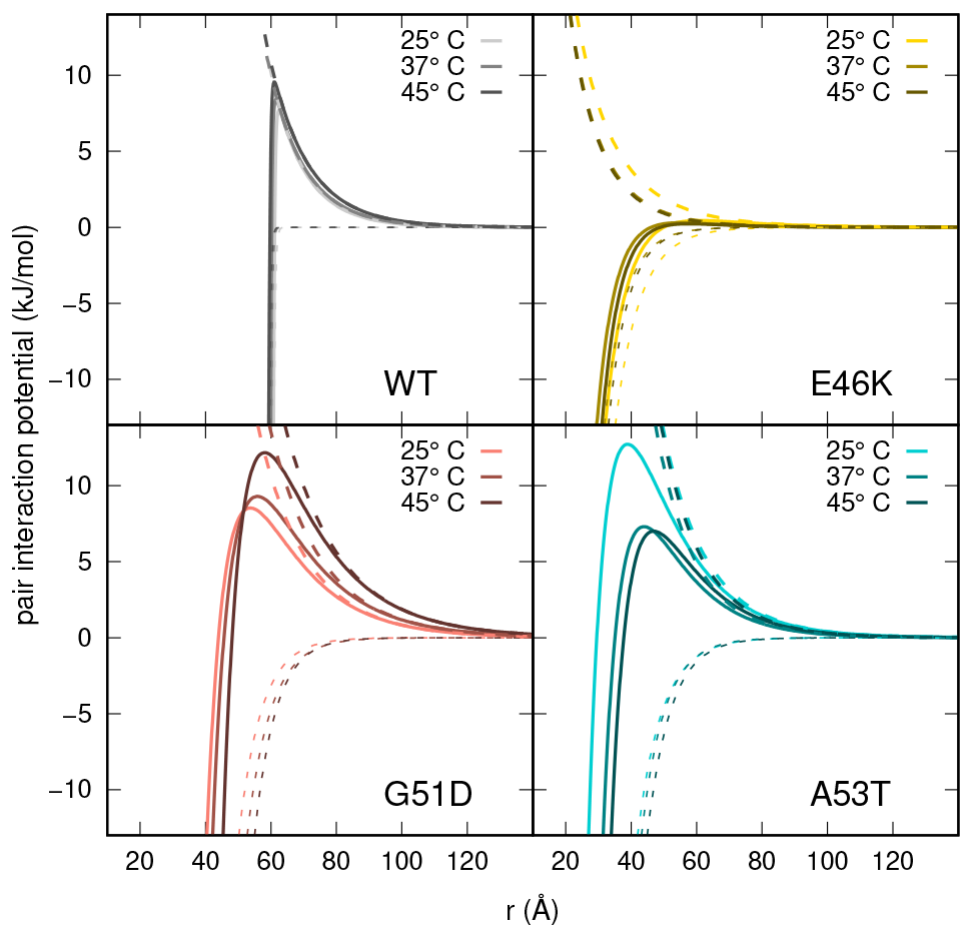
Pair interaction potentials, *u*(*r*) (solid lines), together with repulsive and attractive terms, *u*_*C*_(*r*) (dashed lines) and *u*_*A*_(*r*) (dotted lines), respectively, obtained by SAXS data analysis with VBWSAS for WT α-syn (*c* = 5.5 g/L) and three mutants G51T (*c* = 6.1 g/L), E46K (*c* = 6.0 g/L), and A53T (*c* = 5.4 g/L) at the three temperatures as shown in the legends.

A further indication of the different interaction regime for the E46K α-syn type is shown in Figure 10, which reports the trends of the pair interaction potential *u*(*r*) (solid lines) and its attractive (dashed lines) and repulsive (dotted lines) contributions, corresponding to the cases shown in Figure 9. These results suggest that the monomers of the E46K α-syn type may experience an overall isotropic attraction effect, probably due to the fact that their net number of electric charges, |*Z*_1_|, is ≈7, less than at least 2 units with respect to the other three α-syn types (Table S2, Supporting Information).

Interestingly, the peculiarity of the E46K mutant in terms of net charge impacts also its long term interactions in solution^43^ and on the known structural properties of E46K fibrils.^68^ Indeed, Ranjan and Kumar^43^ showed, using solution NMR, that the substitution of the glutamic acid E46 with a positively charged lysine is the only mutation associated with pathology that present long-range contact rearrangements at the C-terminal of the protein. Coherently, E46K amyloid fibrils show the largest chemical shift perturbations as measured with solid state NMR. Therefore, the E46K mutation determines a substantial change in the fibril structure compared to WT α-syn and other pathological mutants studied by Tuttle et al.^68^

The other important piece of information that can be drawn from the VBWSAS analysis of SAXS data presented here relates to the determination of the *propensity* of each *a*-residue of α-syn, in WT or mutant type, to populate the *r*-region of the Ramachandran map shown in Figure 2, as described in the Propensities section. In fact, this type of information allows one to understand how mutations in a single amino acid impact the propensity to form β-sheets and therefore alter the intermolecular and intramolecular interactions that govern the aggregation properties of the protein.

**Figure 11:**
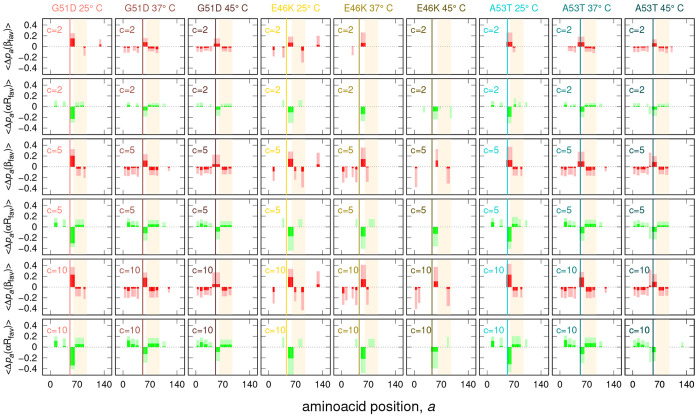
Mean propensity changes between mutant and WT α-syn reported as a function of the amino acid position *a* for the energetically favorable β-sheet region (β_fav_, red histograms) and the energetically favorable right α-helix region (αR_fav_, green histograms). See Figure 2 for the definition of the Ramachandran plot regions. The NAC portion of the sequence is highlighted in light orange. The position of the point mutation is indicated by the vertical line. Concentration, *c*, is expressed in g/L.

According to eq 23, propensities are indeed functions of the average monomer population weights ⟨**w**⟩ and depend on the indexes *r*_*i*,*g*,*a*_ that show, for each *g*-chain of each *i*-conformer of the ensemble, the region of the Ramachandran map to which an *a*-residue belongs. To calculate these indexes, we have determined the ϕ, ψ angles of the residues with the pdbtorsions tool, from the BiopTools package,^69^ applied to all PDB files of our ensemble. Propensities *p*_*a*_(*r*) of the 140 residues of α-syn, in the WT and in the three mutant types, derived by the VBWSAS analysis of our SAXS data, have been calculated for three representative concentrations and three temperatures by using the average monomer population weight values reported in Figure 7. In order to highlight the effect of point mutations, we have considered the differences Δ*p*_*a*_(*r*) between the propensities of the mutant α-syn and the ones of the WT. Moreover, to better identify the role of the point mutation in promoting a significant change of the secondary structure of sufficiently long sequences of residues, we have established a simple criterion, as follows. We consider all the possible sequences of at least eight subsequent residues starting from the *a*_1_-residue and ending in the *a*_2_-residue, with *a*_2_ – *a*_1_ ≥ 7. Then, we check whether the value of Δ*p*_*a*_(*r*) of *all* residues *a* with *a*_1_ ≤ *a* ≤ *a*_2_ have the same positive or negative sign. If the check is validated, the mean change of propensity  and the corresponding standard deviations are assigned to all the residues of the sequence *a*_1_ – *a*_2_, and these values are reported in the histogram. Otherwise, the histogram bars in the range *a*_1_ ≤ *a* ≤ *a*_2_ are fixed to 0. Hence, the lack of bars for a sequence is simply the result of Δ*p*_*a*_(*r*) with the opposite sign within that sequence. A length of eight residues segment was chosen because it approximately represents the average persistence length of a polypeptide.^70^ Resulting histograms of the application of this criterion are reported in Figure 11 for the two most significant regions, β_fav_ (red histograms) and αR_all_ (green histograms).

Looking at the G51D panels of Figure 11, a clear presence of a high red bar can be observed, corresponding to an approximate 0.2 increase of the β_fav_ propensity. At *c* = 2 g/L, this bar is from residue 51 (where the mutation is, evidenced by a thin vertical line) to residue 64, just before the NAC sequence, shown in a light orange background. By increasing *c* at 25 °C, ⟨Δ*p*_*a*_(β_fav_)⟩ increases, and in the middle of the NAC sequence, a bar with negative ⟨Δ*p*_*a*_(β_fav_)⟩ appears, suggesting that in this part of the NAC sequence WT α-syn is more prone to form strands than the G51D type. Concomitantly, in the same sequences, the propensity of αR_all_ decreases by approximately 0.2, suggesting a helical-to-sheet mechanism promoted by the G51D mutation. By increasing *T*, the height of the more intense positive bar decreases, and four more negative bars appear, two on the left and two on the right of the positive bar. At 45 °C, the positive bar is wider, ranging from residue 43 to residue 64. In summary, at the highest *T*, the change in the propensity going from the WT to G51D type, regarding the β_fav_ region, is due to the increase of propensity in the sequence comprised between the residues 43 and 64 in the G51D type and a decrease of the propensity in sequences on the left of residue 43 and on the right of residue 64 and can be attributed to a more marked presence of strand-rich tetramers and a less marked presence of strand-rich trimers in the G51D α-syn type in respect to WT α-syn.

The case of the E46K α-syn type is different and should be discussed by bearing in mind that mostly monomeric conformers are present in solution at any *c* and *T*, as determined by the analysis of ⟨**w**⟩ previously described. The propensity panels regarding E46K in Figure 11 show the presence of a high red bar, in the region of β_fav_, close to the mutation position 46, from residue 51 to residue 65. For *c* = 2 g/L, this bar is more marked at 37 °C and disappears at 45 °C. At *c* = 5 g/L and, more clearly, at *c* = 10 g/L beside this bar, there is a small bar in the NAC sequence with a negative change of propensity. All these red bars compensate with the green bars, indicating, also for this mutant, a helical-to-strand mechanism. In summary, the E46K α-syn type proteins are painted as interacting monomers, and the monomers with highest ⟨*w*_*i*_⟩ are the ones that show a higher β_fav_ propensity in the sequence of residues 51–65.

Finally, we look at the A53T panels of Figure 11, a α-syn mutant type that, according to the analysis of ⟨**w**⟩ previously discussed, is mainly constituted by monomeric (1A-subclass) and tetrameric strand-rich (4E-subclass) conformers. We see a positive red bar, indicating a positive change of propensity in the β_fav_ region of the Ramachandran maps in a sequence close to the residue 53, where the mutation has occurred, ranging from residue 51 to residue 67. At 25 °C, this bar increases with *c*, and on the right, inside the NAC sequence, a negative bar is growing. At 37 °C and for *c* = 2 g/L and *c* = 5 g/L, the positive bar is wider, extending from residue 47 to residue 65, and other negative bars on the left and on the right of this sequence appear. At 37 °C and *c* = 10 g/L, the positive bar returns narrower, from residue 51 to residue 64. Passing at 45 °C, for any *c*, the positive bar remains from residue 51 to residue 64. Hence, if we consider that the wider the sequence with a positive change of β_fav_ propensity is, the higher is the tendency of the mutant to trigger the cross-β nucleation process,^71^ we can conclude that for the A53T α-syn mutant type, the most effective conditions occur at low concentration and 37 °C. This subtle effect could be due to the intricate interplay among the subclasses of conformers weights ω_*m*_ (Figure 8) that show a maximum at around 37 °C for the A53T α-syn mutant type.

For the sake of completeness, in Figures S10–S18 of the Supporting Information, the mean change of propensity for all eight regions of the Ramachandran map are reported.

## Conclusions

The possible presence of folded α-syn tetramers in prefibrillar conditions, together with unfolded monomers, is an issue widely discussed.^8,46−48^ Some experiments have shown that the detection of such tetramers depends not only on the chemical–physical conditions but also on the origin of α-syn, which can be produced using bacteria or isolated from mammalian cells as well as from red blood cells. But unfortunately, there is not a clear reproducibility of these results, and consensus on their interpretation has not been reached yet.^72^ Regarding the use of SAXS techniques, coupled with proper ensembles of conformers, to investigate α-syn in conditions prodromic to formation of fibrils, most of the published results have only considered the radius of gyration calculated in a small range of *q*, typically by Guinier’s approximation.^8,10,73,74^ In other cases, SAXS curves have been analyzed in the full range of *q*, but without considering the absolute calibration of data and so by finding an optimum scaling factor κ or flat background *B*.^31,39^ We did not find studies that considered long-range interactions, which cause, possibly also at low concentration, a broad interference peak that could affect the Guinier’s region.

To the best of our knowledge, this is the first time that an approach is proposed to explore the question of the possible species of α-syn oligomers present at the prefibrillar state. We have fully exploited the performances of one of the most advanced synchrotron SAXS instruments (BM29, ESRF, Grenoble), which allows a precise absolute scale calibration and a perfect buffer subtraction, avoiding the need to use nuisance parameters, such as κ and *B*, in fitting data. Accordingly, the results that we have obtained with the VBWSAS method take into account the modifications provided by *c* and *T* on the absolute scale  in the entire *q* range, and the model we have applied includes the variations of the form factor, based on a thermodynamic scheme, and the ones of the structure factor, described by the well-established PY-RPA approximation. Indeed, our SAXS curves do suggest, before any interpretation, that some oligomeric forms of α-syn could be present in our samples. Despite the fact that the most updated protocols proposed methods to remove the oligomers,^72^ they are the result of an equilibrium process so that oligomers are bound to be naturally present together with monomers.

Results that we have obtained for WT α-syn partially contradict the ones derived by Gurry et al.^45^: the most prominent forms of α-syn are trimers, not tetramers. Among trimers, at low *T*, the most abundant are the helical-rich ones, whereas at high *T* they are the strand-rich ones. Considering the α-syn G51D type, which is one of the most aggressive mutants leading to the earliest onsets of PD disease,^75^ our results indicate that strand-rich tetramers are the most abundant aggregated form of α-syn at all temperatures, whereas any trimeric form has a negligible population. This important result suggests the possibility that strand-rich tetramers can be the multimeric species that trigger the nucleation of fibrils or that these soluble multimers (or the larger ones that can form in the early stages of amyloid fibril formation) may be toxic species for the neurons. Results obtained for the α-syn A53T type, a mutant considered of clinical significance and widely studied,^14^ also confirm the predominance of strand-rich tetramers that tends to diminish with *T*, suggesting that A53T may be less aggressive than G51D in the early onset of fibrillation thus providing further details on nucleation events that occur at the beginning of the aggregation process. The propensity analysis of both G51D and A53T, in comparison with WT α-syn, confirm these interpretations, indicating an increase of the propensity of the β-favored region in the portion of the sequence between the mutation point and the NAC portion. Completely different are the VBWSAS results for the E46K mutant, which is known to provoke small changes in the conformation by enhancing the contacts between N- and C-termini of α-syn.^14^ Our VBWSAS analysis does confirm that this mutant is mostly present as monomeric conformers, with a preference for the ones with the smallest values of *R*_*g*_. Moreover, such monomers show a long-range unspecific tendency to attract themselves. VBWSAS results also show that only at high *T* there is an increase of the propensity of the β-favored region. The higher compactness of the E46K monomers is also confirmed by the maps of C_α_–C_α_ distances reported in Figure S6 of the Supporting Information, showing an average negative difference between pairs of C_α_ atoms between the E46K type and WT in the off diagonal regions. These results are in good agreement with the differences in the aggregation propensities and fibrils structure for the E46K mutant.

Overall, the VBWSAS method applied to α-syn shows evidence that the different aggregation and toxicity behavior of the pathogenic mutants is likely to originate from the different disordered conformers that these protein species naturally populate in prefibrillar conditions. Our results may suggest that the structure of these conformers should be well characterized in order to understand how they contribute to the α-syn aggregation process in relationship to PD etiopathogenesis and features.

Here, α-syn and its mutants have been used as a complex paradigm for IDPs, but the proposed VBWSAS approach can be easily extended to other IDPs whose behavior biochemistry is crucial for understanding the early molecular events that lead to IDP-related neurodegenerative diseases. In summary, we have shown that to disentangle conformational information out of a suitable IDP ensemble by means of SAXS data it is necessary to adopt a method with solid foundations from both statistical and thermodynamic points of view. VBWSAS can serve this aim.

The VBWSAS software is available upon request.

## Supporting Information Available

Variation with *T* of concentration and solvent SLD, protein net charge and its *T*-dependency, SAXS data and best fit, correlation maps of average monomer population weights, maps of C_α_–C_α_ distances, and mean change of propensities (PDF)
