# Supplementary material for: Comprehensive Structural and Thermodynamic Analysis of Prefibrillar WT α-Synuclein and Its G51D, E46K, and A53T Mutants by a Combination of Small-Angle X-ray Scattering and Variational Bayesian Weighting
Source: J Chem Inf Model. 2020 Aug 31;60(10):5265–81. doi: 10.1021/acs.jcim.0c00807 (PMC8154249; doi:10.1021/acs.jcim.0c00807)
Supplement: Supplementary file 1 — ci0c00807_si_001.pdf [file ci0c00807_si_001.pdf]

Supporting Information

Comprehensive Structural and Thermodynamic Analysis of Prefibrillar WT α-Synuclein and Its G51D, E46K, and A53T Mutants by a Combination of Small-Angle X-ray Scattering and Variational Bayesian Weighting

Paolo Moretti[1], Paolo Mariani[1], Maria Grazia Ortore[1], Nicoletta Plotegher[2], Luigi Bubacco[2], Mariano Beltramini[2], Francesco Spinozzi[1*]

[1] Department of Life and Environmental Sciences, Polytechnic University of Marche, 60131 Ancona, Marche, Italy

[2] Department of Biology, University of Padova, 35121 Padova, Veneto, Italy

*f.spinozzi@univpm.it

Variation with T of concentration and solvent SLD

Since we work in dilute conditions, we can assume that the thermal expansivity of the solutions are mostly due to water. Hence, the protein w/v concentration c changes with T according to

$$c(T) = c(T_0) d_{\rm w}(T) \tag{S1}$$

where $d_{\rm w}(T)$ is the bulk water relative mass density in respect to the reference temperature $T_0 = 298.15$ K and $c(T_0)$ is the nominal weight/volume concentration of α-syn samples at T_0. Data of $d_{\rm w}(T)$ derived by Kell[1] have been approximated in our investigated range $25 - 45^\circ$ C by this function of T,

$$d_{\rm w}(T) \quad = \quad e^{-\alpha_{\rm w}(T-T_0)-\beta_{\rm w}(T-T_0)^2/2}. \tag{S2}$$

We found the following optimum value of the thermal expansivity at T_0, $\alpha_{\rm w} = 2.5 \cdot 10^{-4}$ K^{-1}, and its first derivative, $\beta_{\rm w} = 9.8 \cdot 10^{-6}$ K^{-2}.

Table S1: Acidic dissociation constants at T_0, parameter s_i of N-term, C-term and side chain groups and composition of the different α-syn types investigated by SAXS.

i	residue	$\mathrm{p}K_{\mathrm{a},i}(T_0)$	s_i	n_i			
				WT	G51D	E46K	A53T
1	C-term	3.00	-1	1	1	1	1
2	ASP	3.90	-1	6	7	6	6
3	GLU	4.07	-1	18	18	17	18
4	HIS	6.04	$+1$	1	1	1	1
5	N-term	8.00	$+1$	1	1	1	1
6	CYS	8.18	-1	0	0	0	0
7	TYR	10.46	-1	4	4	4	4
8	LYS	10.54	$+1$	15	15	16	15
9	ARG	12.48	$+1$	0	0	0	0
10	SER	14.20	-1	4	4	4	4
11	THR	15.00	-1	10	10	10	11

On the same basis, the bulk water scattering length density used in the SASMOL[2] method is calculated with the following expression

$$\rho_0 = r_{\mathrm{e}} n_{\mathrm{e,w}} \rho^{\circ}_{m,\mathrm{w}} N_A d_{\mathrm{w}}(T)/M_{\mathrm{w}} \tag{S3}$$

where $r_{\mathrm{e}} = 0.28 \cdot 10^{-12}$ cm is the classical radius of the electron, $n_{\mathrm{e,w}} = 10$ is the number of electrons of the water molecule, $\rho^{\circ}_{m,\mathrm{w}}$ the water mass density at T_0 and M_{w} the water molecular mass.

Protein net charge and its T-dependency

The monomer net protein charge Z_1 has been calculated as a function of pH and temperature and on the basis of the primary sequence of the α-syn type according to the following approximation

$$Z_1(T) \quad = \quad \sum_{i=1}^{11} n_i s_i 10^{-s_i(\mathrm{pH}-(T_0/T)\mathrm{p}K_{\mathrm{a},i}(T_0))} \tag{S4}$$

where 11 acidic groups have been considered, the N-term, the C-term and the 9 side chain groups listed in Table S1, together with their acidic dissociation constants at T_0, $\mathrm{p}K_{\mathrm{a},i}(T_0)$. n_i is the number of groups in the primary sequence and the parameter s_i could assume a value of -1 or $+1$ on the basis of the acidic or basic nature of the group, respectively. Results at pH = 7 and at the temperatures of SAXS data for the various α-syn types are reported in Table S2.

Table S2: Net charge a different T of the investigated α-syn species.

α-syn	$Z_1(25°)$	$Z_1(37°)$	$Z_1(45°)$
WT	−9.0	−9.1	−9.2
G51D	−10.0	−10.1	−10.2
E46K	−7.0	−7.1	−7.2
A53T	−9.0	−9.1	−9.2

SAXS data and best fit

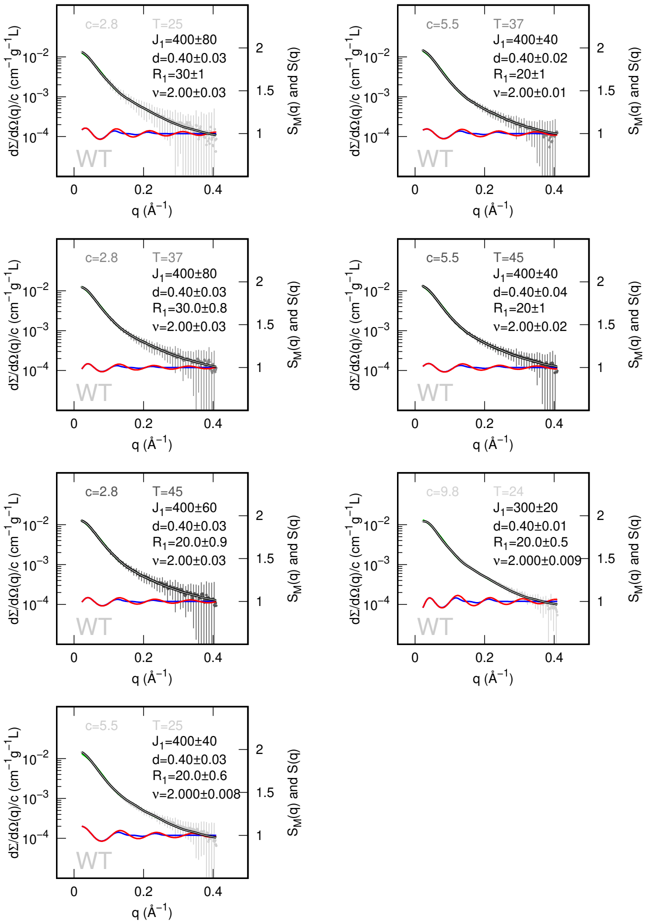

Figure S1: Panels reporting each experimental SAXS curve (points) measured for the WT α-syn and the best fit obtained with VBWSAS (solid black and white line). The average form factor $P(q)$, according to Eq. 17 is the solid green curve. By referring to the right y axis, the protein-protein structure factor $S(q)$ and the measured structure factor $S_M(q)$ are reported as solid red and blue curves, respectively (Eq. 18). Concentration and temperature is reported on the top of each panel, together with the optimum single curve (regularized) parameters related to the structure factor: J_1 (in kJ/mol), d (in Å), R_1 (in Å) and ν.

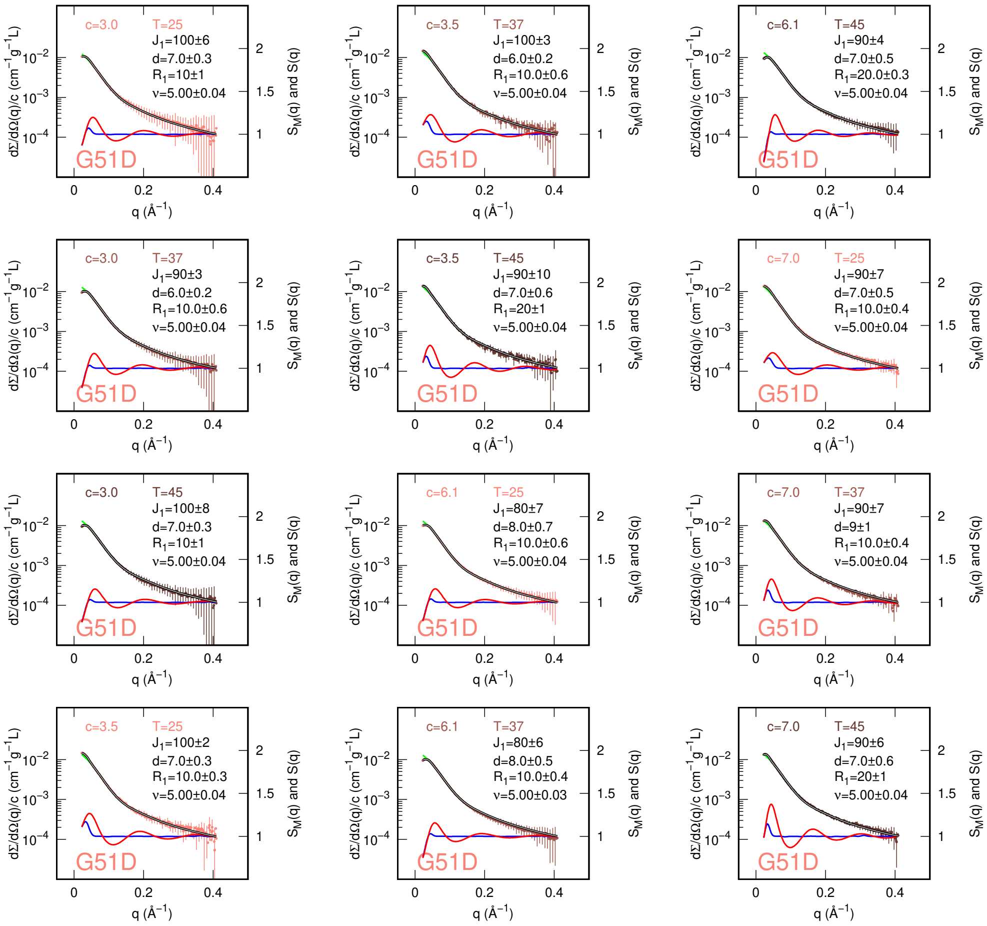

Figure S2: Panels reporting each experimental SAXS curve (points) measured for the G51D α-syn mutant and the best fit obtained with VBWSAS (solid black and white line). See caption of Fig. S1 for details.

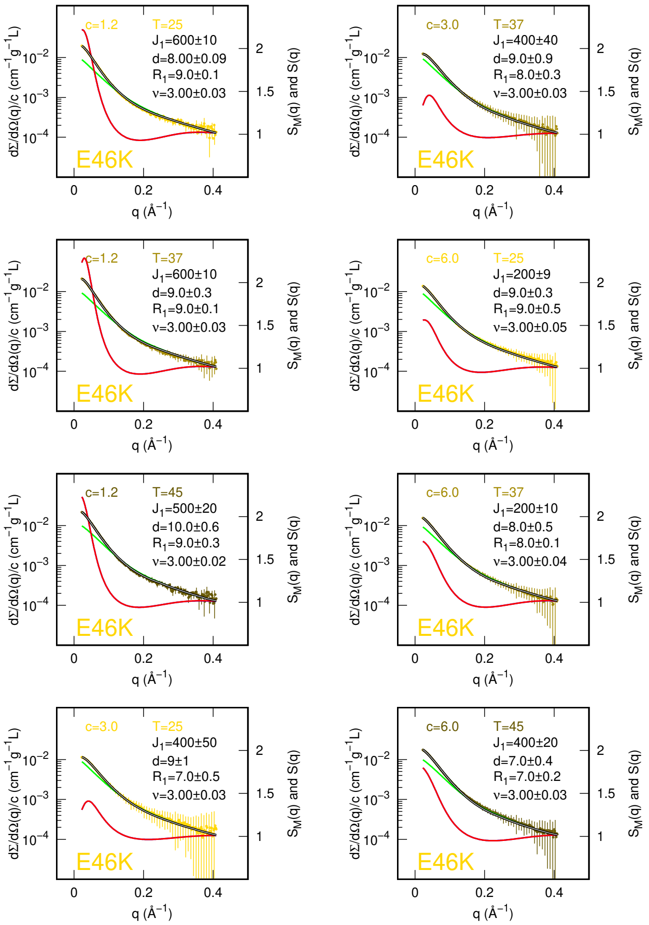

Figure S3: Panels reporting each experimental SAXS curve (points) measured for the E46K α-syn mutant and the best fit obtained with VBWSAS (solid black and white line). See caption of Fig. S1 for details.

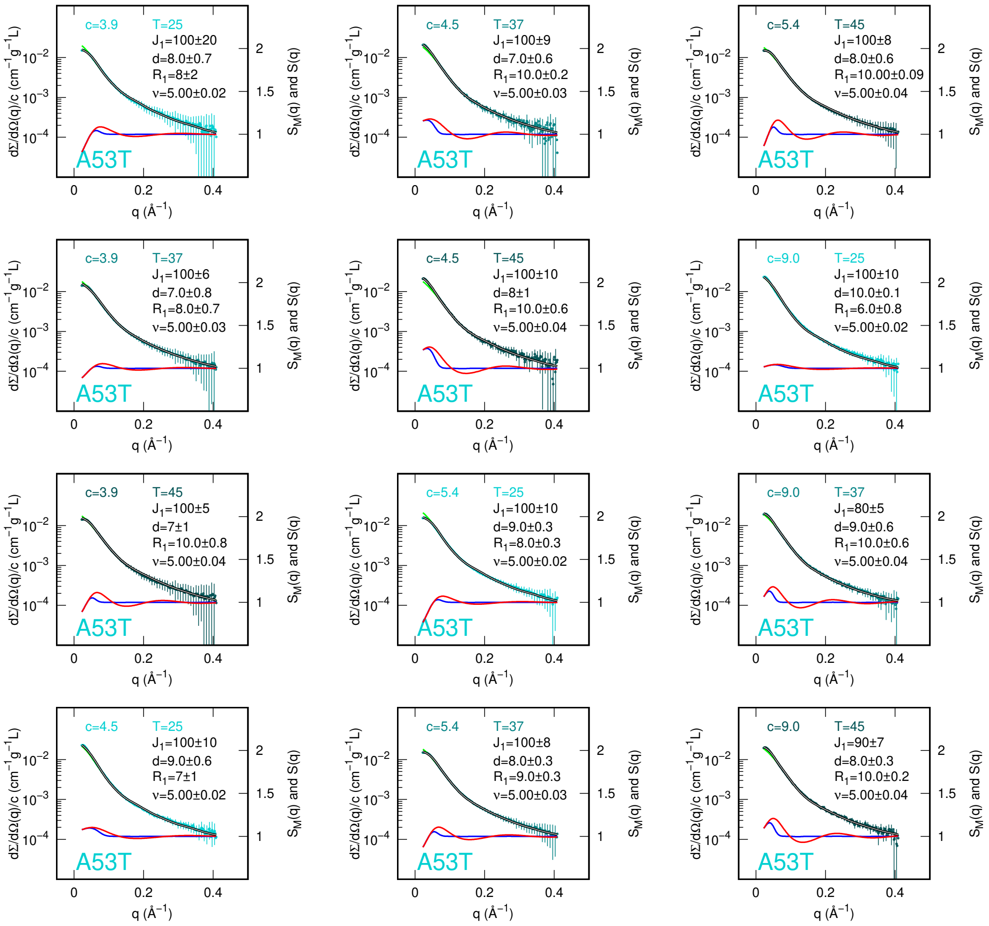

Figure S4: Panels reporting each experimental SAXS curve (points) measured for the A53T α-syn mutant and the best fit obtained with VBWSAS (solid black and white line). See caption of Fig. S1 for details.

Most populated conformers

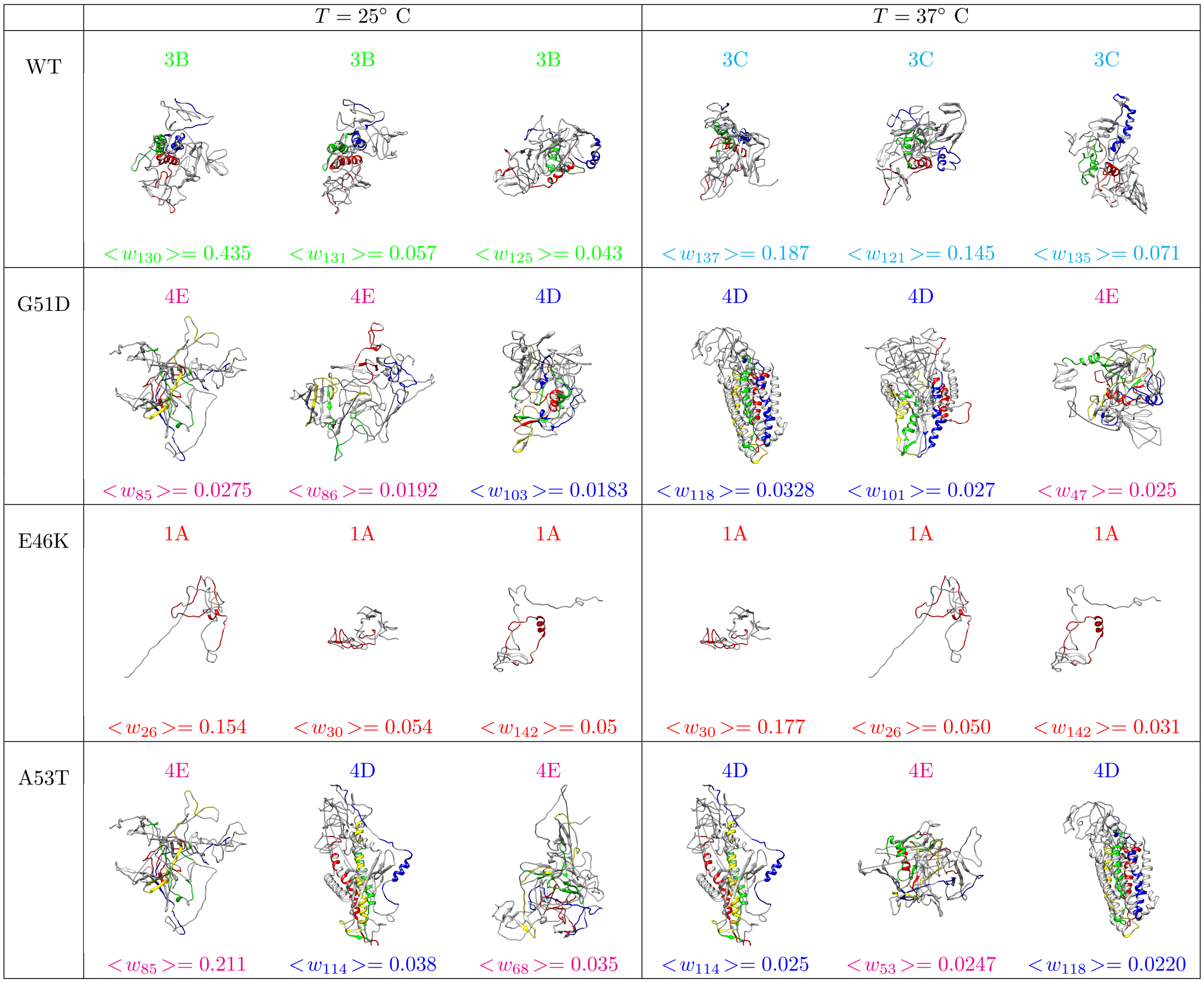

Figure S5: Representations of the first three most populated conformers obtained, for WT α-syn and the point mutant-types G51T, E46K and A53T, by VBWSAS at $c = 10$ g/L, compared at the two temperatures $T = 25°$ C (second column) and $T = 37°$ C (third column). On the top and on the bottom of each conformer, the subclass and the obtained $<w_i>$ (written up to the last significant digit) are reported, respectively.

Correlation maps of average monomer population weights

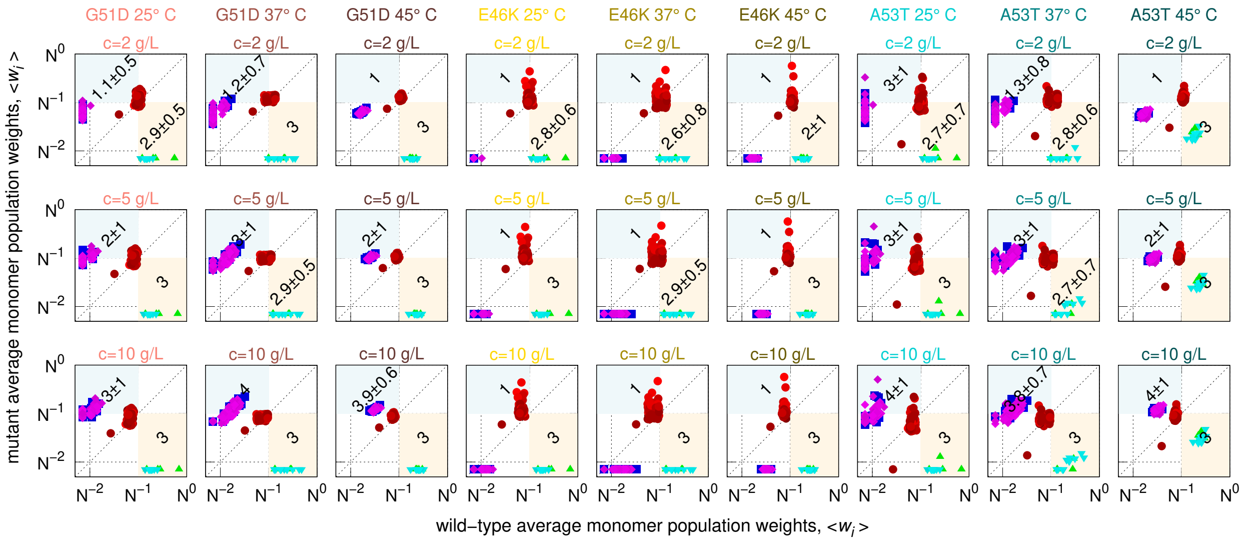

Figure S6: Correlation map of the average monomer population weights of the $N = 189$ conformers between WT α-syn (horizontal axis) and the three mutants G51D, E46K and A53T (vertical axis). Each symbol represents a conformer and is colored on the basis of the its subclass and its radius of gyration, following the same color settings of Fig. 4. Moreover, symbols are assigned according to the classification of Gurry et al.[3]. Circles: monomers (A); down-sided triangles: helical-rich trimers (B); up-sided triangles strand- rich trimers (C); squares: helical-rich tetramers (D); diamonds: strand-rich tetramers (E). The light-blue (light-orange) off-diagonal quadrant indicates the group of conformations with $<w_i>$ simultaneously greater (lower) than N^{-1} for the mutant and lower (greater) than N^{-1} for WT. In each off-diagonal quadrant the average values $<m>$ of the group of conformations present in the quadrant are reported as tilted labels. Conformations with $<w_i> < N^{-2}$ for mutant-type and WT are shown in the middle of the left and bottom strips, respectively.

Maps of $C_\alpha - C_\alpha$ distances

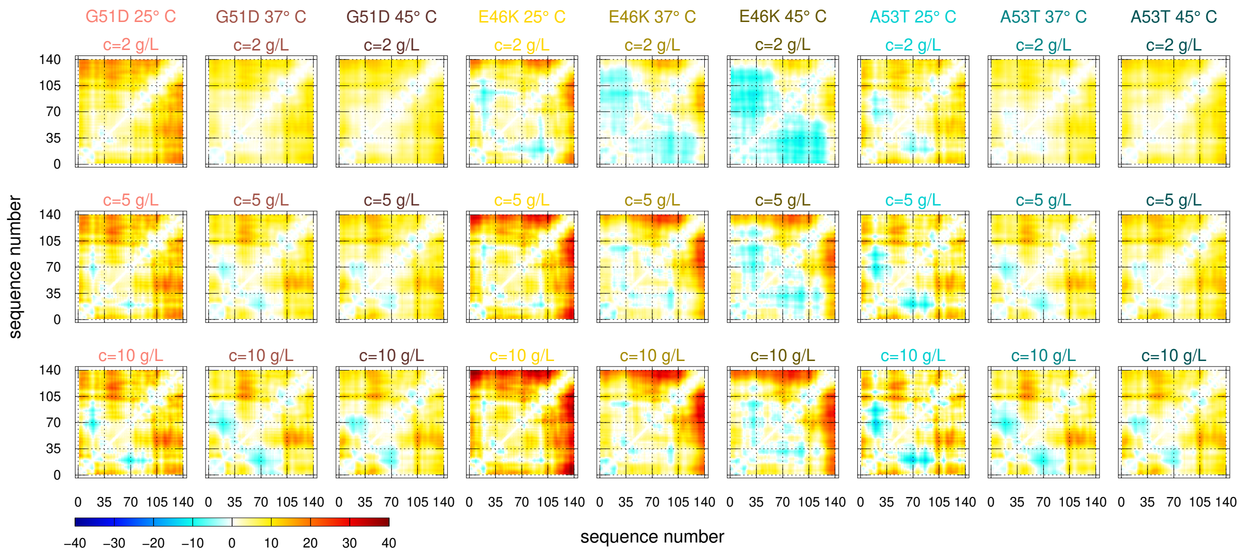

Figure S7: Distance heat map comparing the mean distances between pairs of C_α atoms between α-syn mutant-type and α-syn WT. Color coded is the difference (expressed in Å) of $C_\alpha - C_\alpha$ distances, $d_{a_1 a_2} = < r_{a_1 a_2} >_{\mathrm{mut}} - < r_{a_1 a_2} >_{\mathrm{WT}}$, where $r_{a_1 a_2}$ denotes the mean distance of the C_α atoms of residues a_1 and a_2.

Table S3: Parameters related to the structure factors shown in Fig. 9.

		WT	G51D	E46K	A53T
c	(g/L)	5.5	6.1	6.0	5.4
T	(°C)	25			
$<m>$	(Å)	2.34 ± 0.05	2.15 ± 0.03	1	2.45 ± 0.07
Z_1	(e)	-9.0	-10.0	-7.0	-9.0
Z	(e)	-21.0	-21.5	-7.0	-22.0
R_1	(Å)	22.4 ± 0.6	13.4 ± 0.6	8.6 ± 0.5	8.1 ± 0.3
R	(Å)	29.8 ± 0.8	17.3 ± 0.7	8.6 ± 0.5	10.9 ± 0.4
J_1	(kJ/mol)	370 ± 40	82 ± 7	182 ± 9	126 ± 10
J	(kJ/mol)	660 ± 70	140 ± 10	182 ± 9	230 ± 20
d	(Å)	0.38 ± 0.03	7.9 ± 0.7	9.4 ± 0.3	9.2 ± 0.3
ν		1.810 ± 0.008	5.00 ± 0.04	2.70 ± 0.05	4.99 ± 0.02
T	(°C)	37			
$<m>$	(Å)	2.12 ± 0.02	2.14 ± 0.04	1	2.44 ± 0.02
Z_1	(e)	-9.1	-10.1	-7.1	-9.1
Z	(e)	-19.3	-21.6	-7.1	-22.2
R_1	(Å)	23 ± 1	14.2 ± 0.4	8.2 ± 0.1	9.2 ± 0.3
R	(Å)	29 ± 2	18.3 ± 0.5	8.2 ± 0.1	12.5 ± 0.4
J_1	(kJ/mol)	390 ± 40	79 ± 6	190 ± 10	110 ± 8
J	(kJ/mol)	640 ± 70	130 ± 10	190 ± 10	200 ± 10
d	(Å)	0.36 ± 0.02	8.3 ± 0.5	7.6 ± 0.5	8.4 ± 0.3
ν		1.82 ± 0.01	4.99 ± 0.03	2.71 ± 0.04	4.99 ± 0.03
T	(°C)	45			
$<m>$	(Å)	2.01 ± 0.03	2.13 ± 0.02	1	2.33 ± 0.03
Z_1	(e)	-9.2	-10.2	-7.2	-9.2
Z	(e)	-18.5	-21.8	-7.2	-21.4
R_1	(Å)	23 ± 1	15.5 ± 0.3	7.3 ± 0.2	10.15 ± 0.09
R	(Å)	29 ± 2	19.9 ± 0.4	7.3 ± 0.2	13.5 ± 0.1
J_1	(kJ/mol)	410 ± 40	85 ± 4	360 ± 20	107 ± 8
J	(kJ/mol)	660 ± 70	141 ± 7	360 ± 20	190 ± 10
d	(Å)	0.38 ± 0.04	7.4 ± 0.5	7.3 ± 0.4	8.3 ± 0.6
ν		1.84 ± 0.02	5.00 ± 0.04	2.70 ± 0.03	5.00 ± 0.04

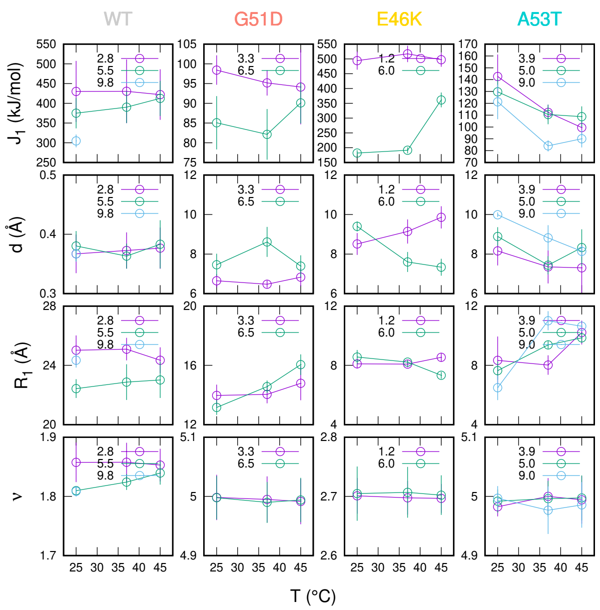

Figure S8: Temperature trends of the fitting parameters related to the structure factor subjected to the regularization procedure (Eq. 26). At any temperature, results obtained for α-syn concentrations within three ranges, low ($c \leq 4$ g/L), middle ($4 < c < 8$ g/L) and high ($c \geq 8$ g/L), are averaged.

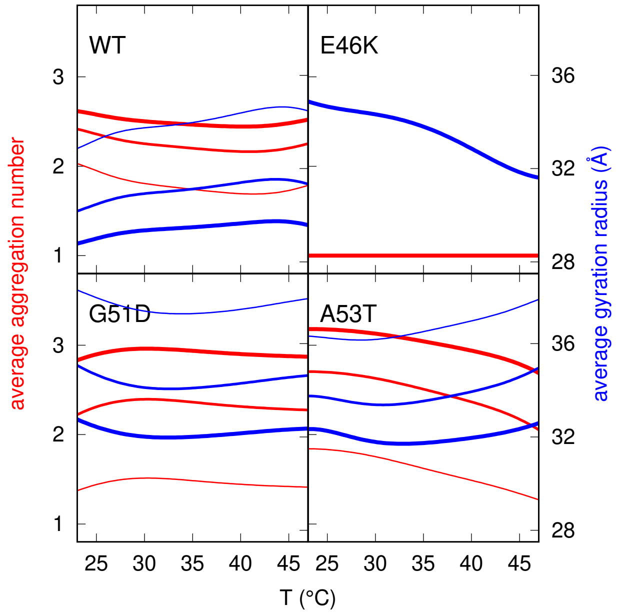

Figure S9: Temperature trends of the average aggregation numbers, $<m>$ (red lines, referred to the left y-axis) and the average radius of gyration, $<R_g>$ (blue lines, referred to the right y-axis) calculated from the thermodynamic parameters found by the VBWSAS analysis of SAXS data for WT α-syn and the three mutants G51T, E46K and A53T.

Mean change of propensities

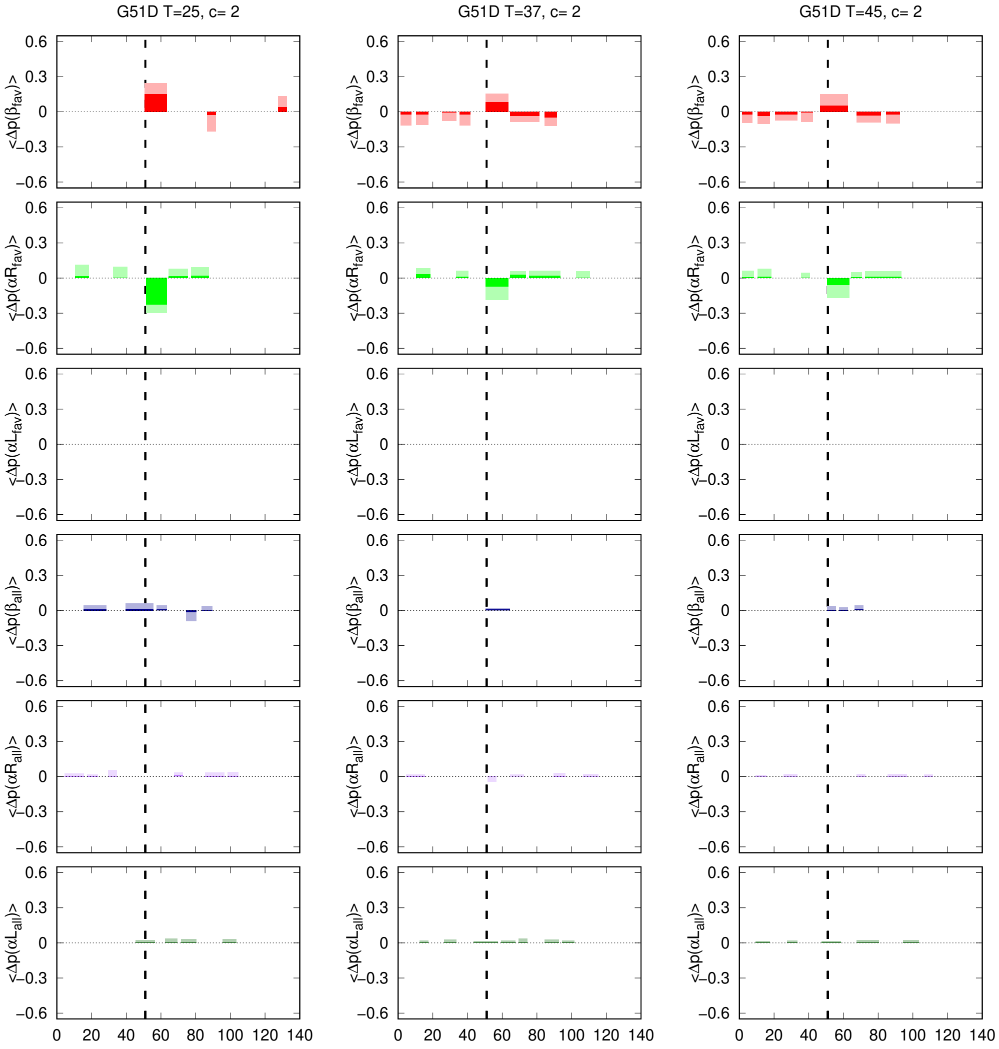

Figure S10: Mean change of propensities between α-syn G51D mutant and WT obtained by the VBWSAS analysis of SAXS data. $c = 2$ g/L.

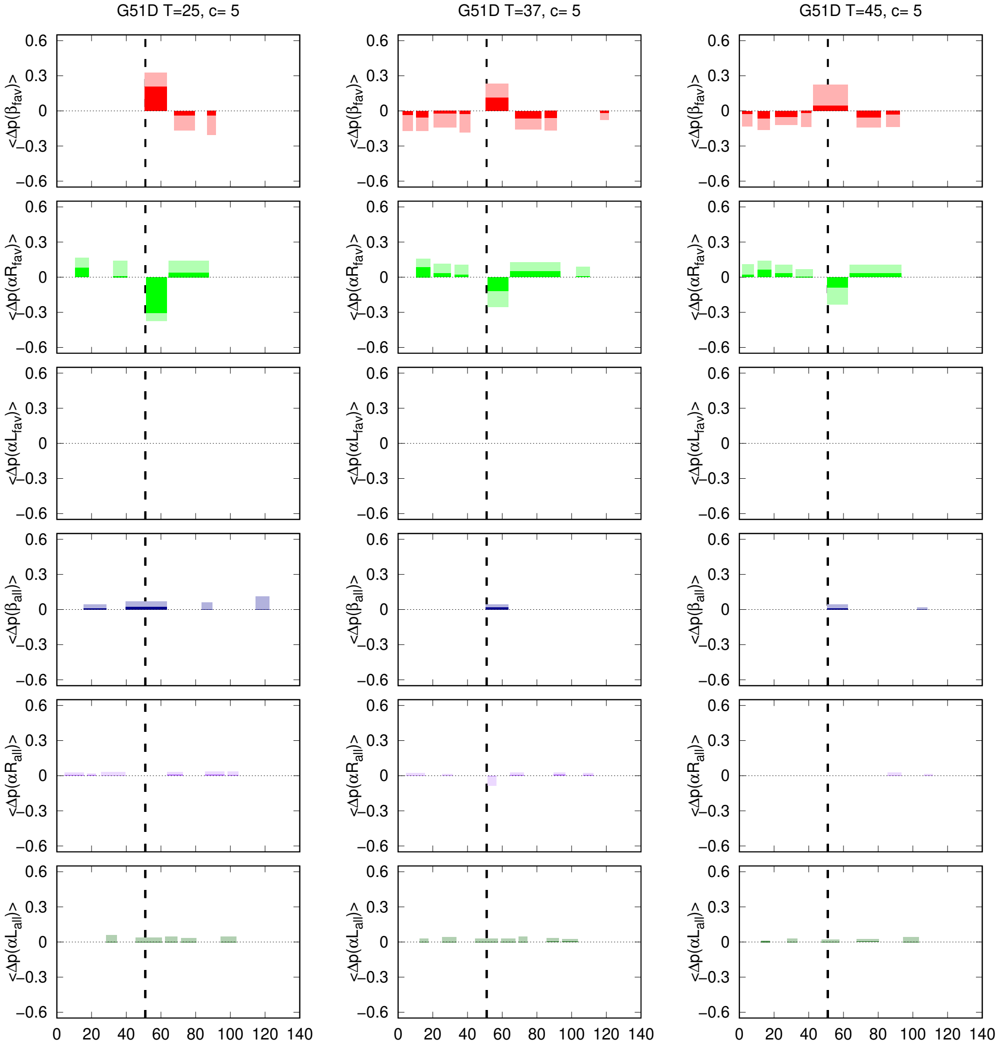

Figure S11: Mean change of propensities between α-syn G51D mutant and WT obtained by the VBWSAS analysis of SAXS data. $c = 5$ g/L.

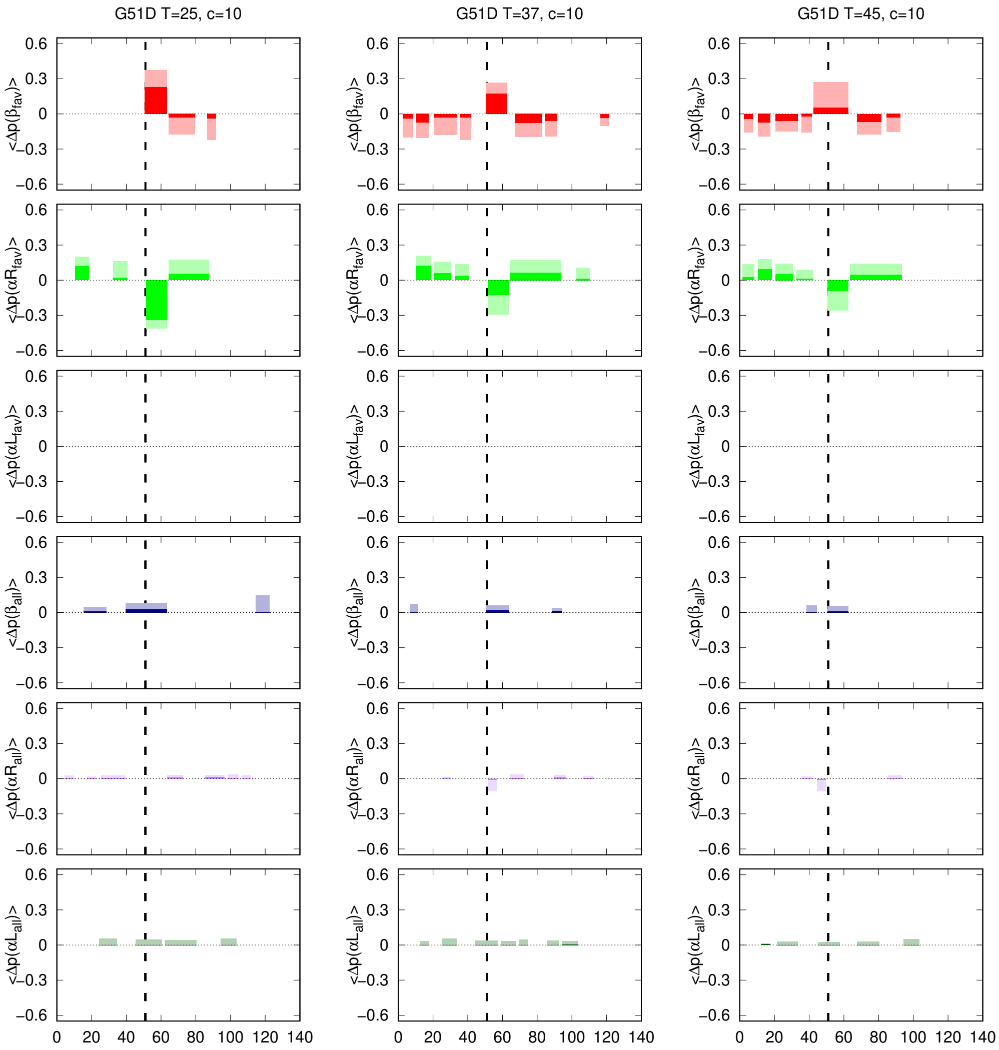

Figure S12: Mean change of propensities between α-syn G51D mutant and WT obtained by the VBWSAS analysis of SAXS data. $c = 10$ g/L.

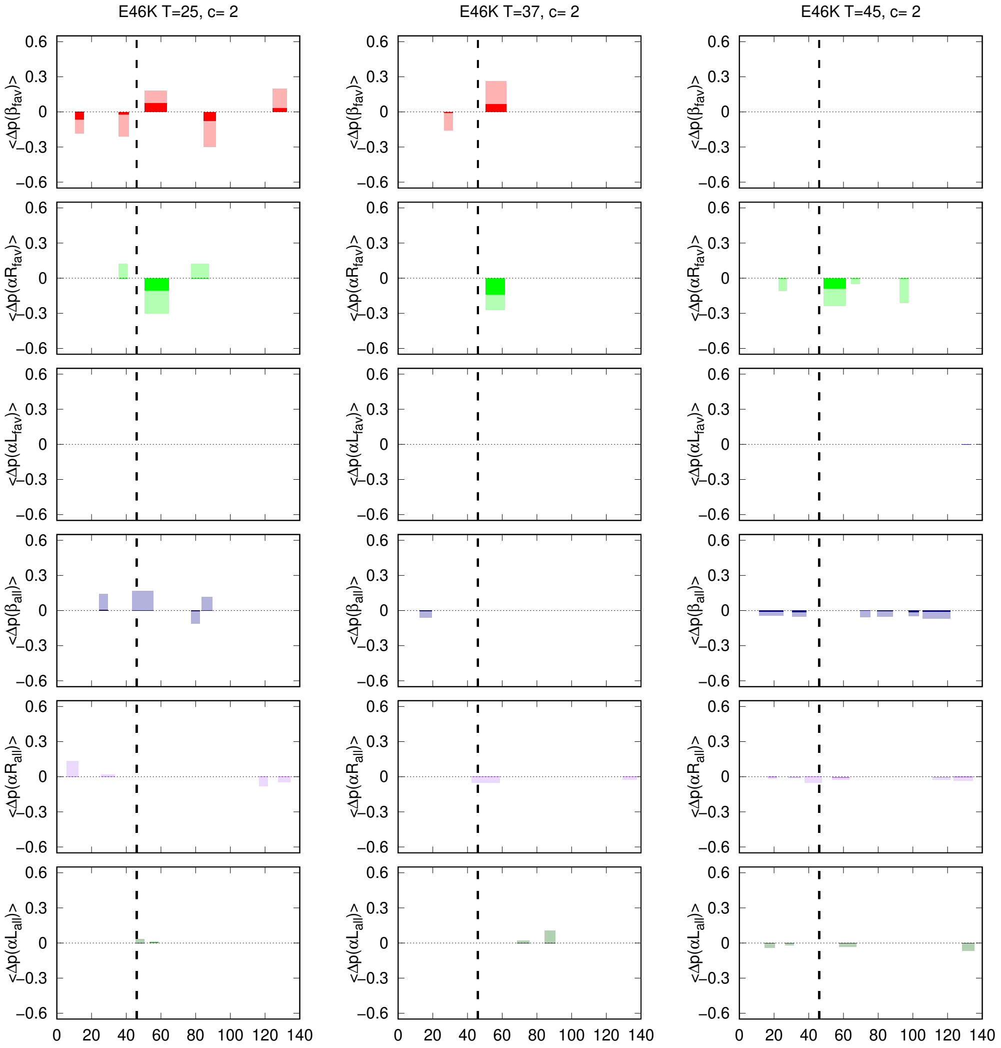

Figure S13: Mean change of propensities between α-syn E46K mutant and WT obtained by the VBWSAS analysis of SAXS data. $c = 2$ g/L.

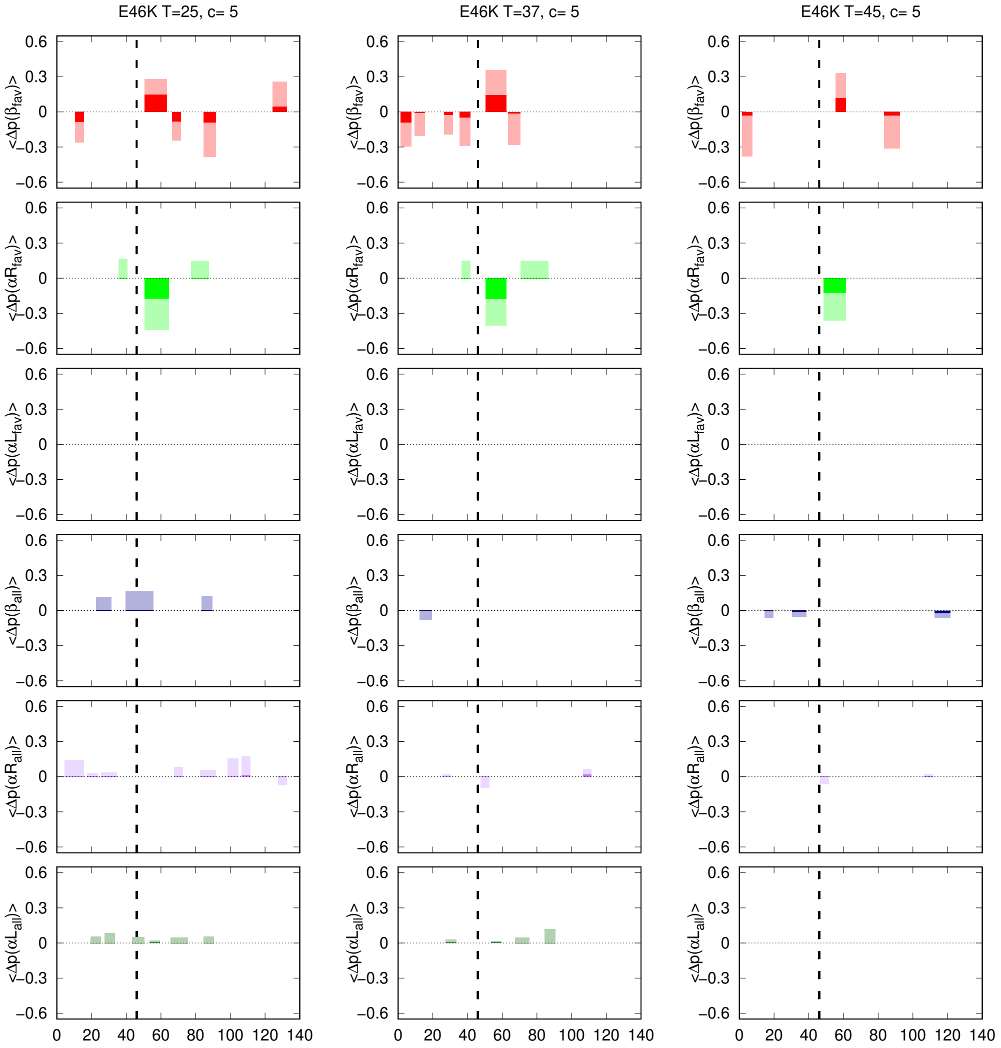

Figure S14: Mean change of propensities between α-syn E46K mutant and WT obtained by the VBWSAS analysis of SAXS data. $c = 5$ g/L.

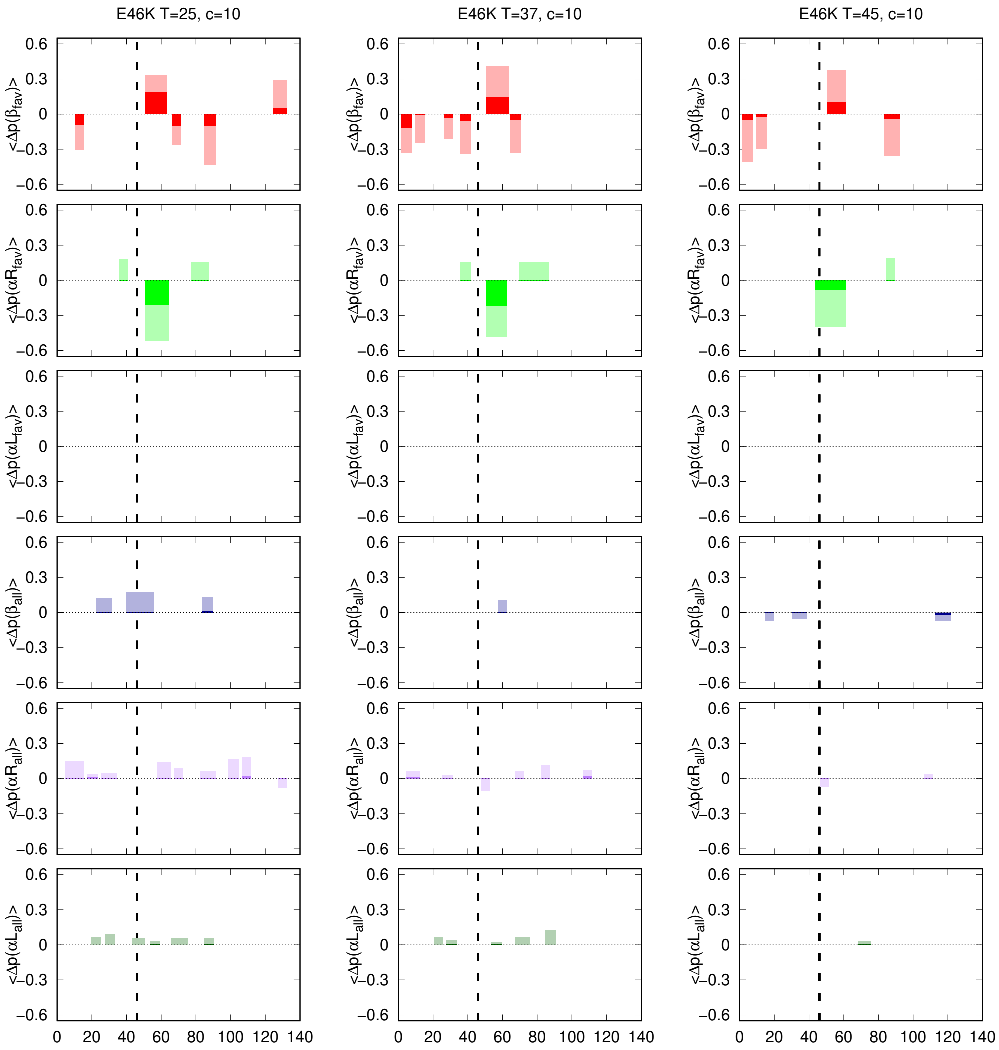

Figure S15: Mean change of propensities between α-syn E46K mutant and WT obtained by the VBWSAS analysis of SAXS data. $c = 10$ g/L.

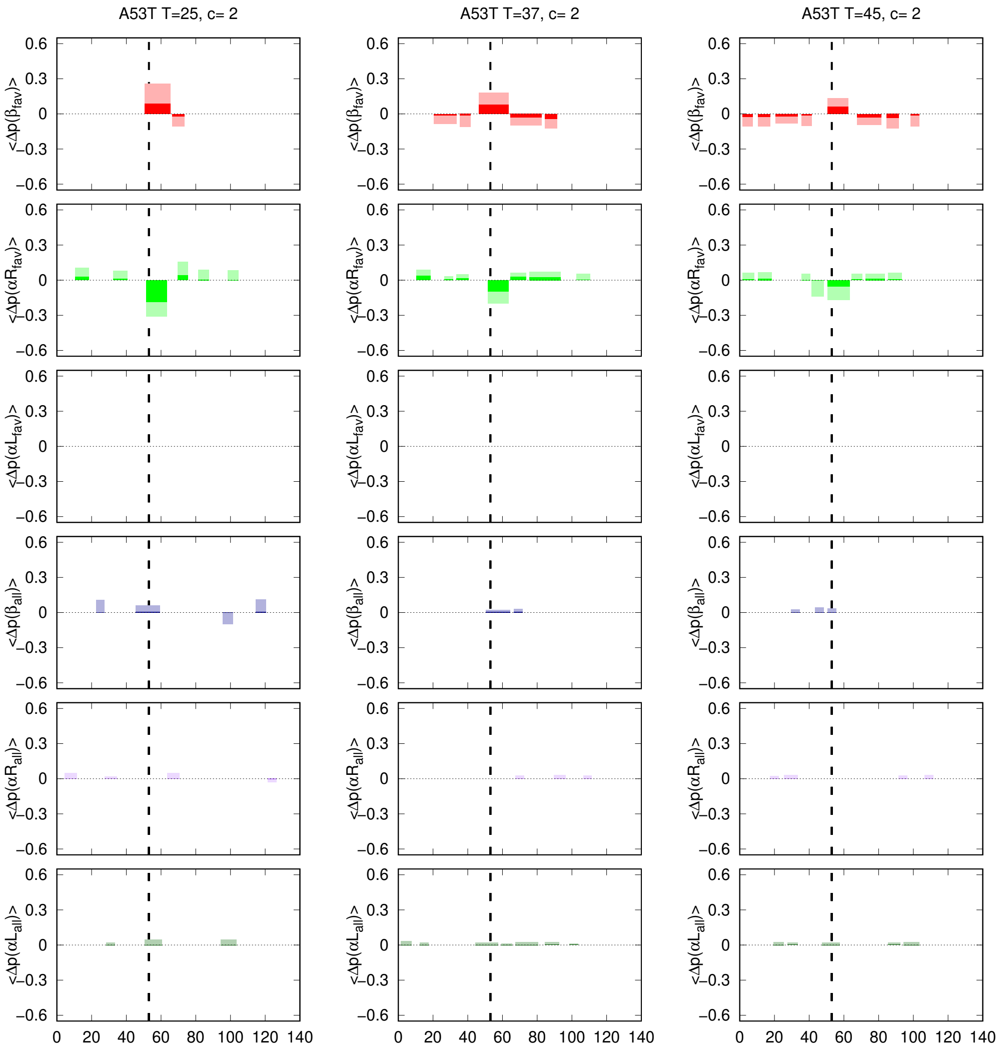

Figure S16: Mean change of propensities between α-syn A53T mutant and WT obtained by the VBWSAS analysis of SAXS data. $c = 2$ g/L.

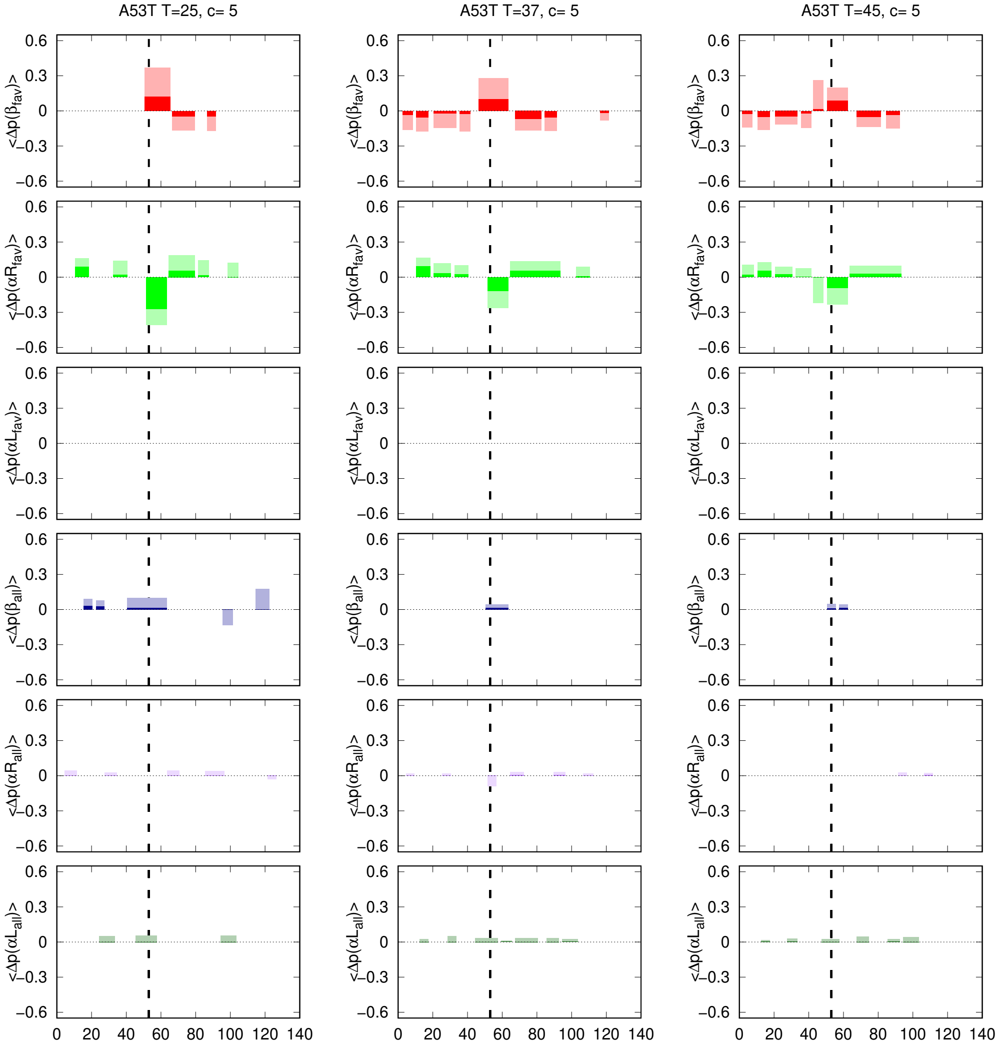

Figure S17: Mean change of propensities between α-syn A53T mutant and WT obtained by the VBWSAS analysis of SAXS data. $c = 5$ g/L.

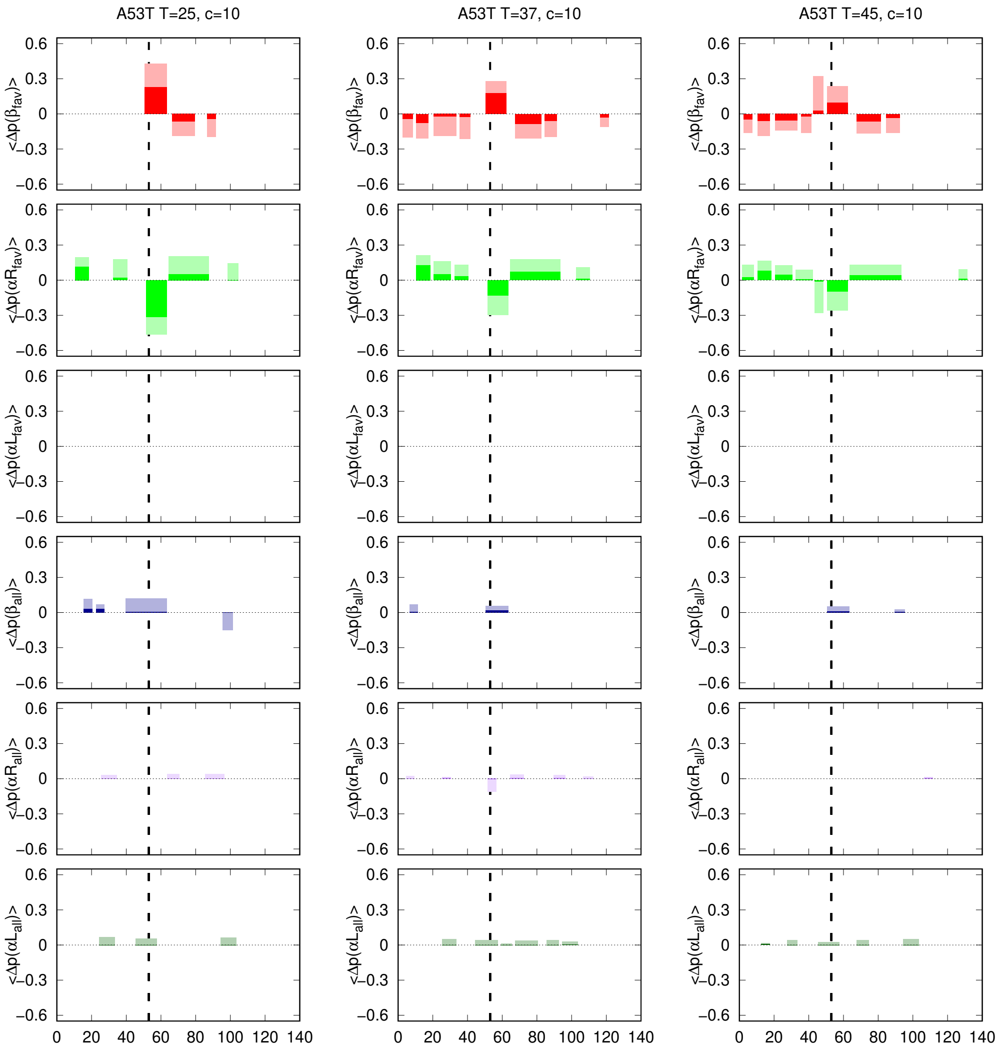

Figure S18: Mean change of propensities between α-syn A53T mutant and WT obtained by the VBWSAS analysis of SAXS data. $c = 10$ g/L.

References

[1] Kell, G. S. Density, Thermal Expansivity, and Compressibility of Liquid Water from 0° to 150°. Correlations and Tables for Atmospheric Pressure and Saturation Reviewed and Expressed on 1968 Temperature Scale. *J. Chem. Eng. Data* **1975**, *20*, 97–105.

[2] Ortore, M. G.; Spinozzi, F.; Mariani, P.; Paciaroni, A.; Barbosa, L. R. S.; Amenitsch, H.; Steinhart, M.; Ollivier, J.; Russo, D. Combining Structure and Dynamics: Non-Denaturing High-Pressure Effect on Lysozyme in Solution. *J. R. Soc. Interface* **2009**, *6*, S619–S634.

[3] Gurry, T.; Ullman, O.; Fisher, C.; Perovic, I.; Pochapsky, T.; Stultz, C. The Dynamic Structure of α-Synuclein Multimers. *J. Am. Chem. Soc.* **2013**, *135*, 3865–3872.
